# Differences in cerebral haemodynamics associated with long-term endurance training in older adults

**DOI:** 10.1162/IMAG.a.1427

**Published:** 2026-11-04

**Authors:** Jack Feron, Foyzul Rahman, Sindre H. Fosstveit, Kelsey E. Joyce, Yifan Gou, Benjamin Talks, Hanzhang Lu, Hilde Lohne-Seiler, Sveinung Berntsen, Carl-Johan Boraxbekk, Karen J. Mullinger, Katrien Segaert, Samuel J.E. Lucas

**Affiliations:** School of Sport, Exercise and Rehabilitation Sciences, University Birmingham, Birmingham, United Kingdom; Centre for Human Brain Health, University Birmingham, Birmingham, United Kingdom; School of Psychology, University Birmingham, Birmingham, United Kingdom; School of Sport, Exercise, and Health Sciences, Loughborough University, Loughborough, United Kingdom; Department of Sport Science and Physical Education, University of Agder, Kristiansand, Norway; Department of Radiology, Johns Hopkins University School of Medicine, Baltimore, MD, United States; Translational and Clinical Research Institute, Newcastle University, Newcastle, United Kingdom; Research Unit, Sorlandet Hospital, Kristiansand, Norway; Institute for Clinical Medicine, Faculty of Medical and Health Sciences, University of Copenhagen, Copenhagen, Denmark; Institute of Sports Medicine Copenhagen (ISMC) and Department of Neurology, Copenhagen University Hospital Bispebjerg, Copenhagen, Denmark; Sir Peter Mansfield Imaging Centre, School of Physics and Astronomy, University of Nottingham, Nottingham, United Kingdom

**Keywords:** cerebral blood flow, arterial transit time, cerebral oxygen extraction, exercise training, cognitive function, arterial spin labelling

## Abstract

Regular exercise can benefit cognitive functions in later life, but the mediating mechanisms remain unclear. Changes to brain vascular health are hypothesised to underpin training-induced brain health benefits, yet how long-term exercise training impacts cerebral haemodynamics is poorly understood. Therefore, this pre-registered cross-sectional study compared cerebral haemodynamics and cognitive functions between untrained (self-report <150 minutes/week moderate-intensity exercise) and long-term endurance trained (≥10 years) older adults (*n* = 28 per group, respective age = 66.0 ± 5.3 and 66.2 ± 5.2 years, respective peak oxygen consumption (V̇O_2peak_) = 28.4 ± 4.7 and 42.3 ± 7.2 mL/kg/min). Multiple post-labelling delay arterial spin labelling (ASL) data were used to estimate cerebral blood flow (CBF) and arterial transit time (ATT) in grey matter at rest. Furthermore, in trained participants, global cerebral oxygen extraction fraction (OEF) was estimated using T_2_-relaxation-under-spin-tagging (TRUST) data (*n* = 26). There were no significant between-group differences (trained – untrained) in global CBF (5.6 [-2.6, 13.9] mL/100 g/min, *P* = 0.197), but global ATT was shorter in trained participants (-0.11 [-0.21, -0.02] seconds, *P* = 0.031). Within regions fed by anterior cerebral arteries, CBF was greater in trained participants (15.8 [3.2, 29.1] mL/100 g/min), but the effect did not survive multiple comparison adjustment (*P*_adjusted_ = 0.064). Cognitive functions did not differ between the groups and were not associated with cerebral haemodynamics. OEF was not associated with V̇O_2peak_ in trained participants, but was strongly inversely associated with global CBF (β = -0.96 [-1.21, -0.75], *P* = 0.002). In conclusion, older adults engaging with long-term endurance training have shorter ATT, counteracting normal age-related ATT lengthening. Longer-term longitudinal studies are required to confirm training-induced regional CBF elevations, which would also counteract normal age-related CBF declines. There was no evidence of relationships with cognitive functions, although the sample was predominantly healthy, educated, and aged 60–70 years. Using multiple post-labelling delays is crucial for future ASL research to isolate CBF and ATT from one another, improving estimation accuracy and delineating between different age-related cerebrovascular mechanisms.

## 1 Introduction

Most cognitive functions worsen with age (Gorbach et al., 2017; Zaninotto et al., 2018), with some evidence indicating simultaneous declines across domains (Tucker-Drob et al., 2019, 2022), which can limit quality of life and independent living (T. Salthouse, 2012; Stites et al., 2018). However, considerable individual variability exists among healthy older adults’ cognitive ageing trajectories (Gorbach et al., 2017; Rodrigues et al., 2025; Zaninotto et al., 2018), with some ageing better than others. Long-term exercise engagement, a modifiable lifestyle factor, has been linked to cognitive benefits in older adults (Barha et al., 2017; Josefsson et al., 2012; M. Zhang et al., 2023). These benefits are hypothesised to be partially mediated by exercise training-induced changes to cerebral haemodynamics (Bliss et al., 2021; Cabral et al., 2019). Indeed, cerebral haemodynamics have been associated with cognitive trajectories in older adults (Anderle et al., 2025; Ebenau et al., 2023; van Dinther et al., 2023; Wolters et al., 2017). However, robust evidence for exercise training-induced changes to cerebral haemodynamics remains elusive, possibly relating to the relatively short-term training interventions (i.e., ≤6 months) commonly studied or the imaging technique used. Therefore, the present study used multiple post-labelling delay arterial spin labelling (ASL) to assess differences in cerebral haemodynamics at rest cross-sectionally between long-term endurance exercise trained (≥10 years) and healthy, untrained older adults, including investigating associations with cognitive functions.

Previous studies investigating responses of CBF at rest to exercise training in healthy older adults have primarily used short-to-medium length interventions, with mixed results. For example, short-term interventions (i.e., 8–12 weeks) have reported both increases and decreases in CBF (Chapman et al., 2013; Kleinloog et al., 2019; Maass et al., 2015), but these findings were limited to small regions or clusters within the brain. A 6-month aerobic intervention also reported no changes to whole-brain CBF (Flodin et al., 2017); however, our 6-month high-intensity exercise intervention unexpectedly induced global grey matter CBF reductions in participants with high (>2 mL/kg/min) cardiorespiratory fitness gains (Feron, Rahman, et al., 2024). Conversely, a longer 1-year intervention, also involving high-intensity exercise, reported an increase in global volumetric CBF (Tomoto et al., 2023). Discrepancies in results are likely attributable to differences in CBF measurement technique, the population, the exercise intervention (i.e., duration/intensity/volume), and the magnitude of cardiorespiratory fitness improvements. Theoretically, more robust conclusions could be made by using longer-term interventions (i.e., years to decades), although these involve considerable logistical and financial challenges. Complimentary information comes from cross-sectional studies that have compared long-term endurance trained middle-aged (Sugawara et al., 2020; Tarumi et al., 2013) or older (Burley et al., 2021; Thomas et al., 2013) adults with sedentary peers, also with mixed results, reporting no absolute CBF differences (Burley et al., 2021; Sugawara et al., 2020; Thomas et al., 2013) or greater occipitoparietal CBF in trained participants (Tarumi et al., 2013). However, data also indicate that relative to young adults, trained older adults may experience less age-related CBF reductions (Sugawara et al., 2020; Thomas et al., 2013).

Of the discussed studies, all except two (Maass et al., 2015; Tomoto et al., 2023) estimated CBF using ASL, but only two of these (Burley et al., 2021; Feron, Rahman, et al., 2024) used multiple-delay ASL—improving CBF estimation accuracy by adjusting for individual and regional differences in arterial transit time (ATT) (Dai et al., 2017; Woods et al., 2024). ATT is the time taken for blood to travel from large cerebral arteries (i.e., internal carotid and basilar) into the cerebral tissue, which lengthens with age (Damestani et al., 2023), disease (McFadden et al., 2025; Sun et al., 2022; Yu et al., 2022), and may also be affected by cardiorespiratory fitness, body mass index, and blood pressure (Feron, Segaert, et al., 2024; Yetim et al., 2023). Consequently, interpretations from previous single-delay ASL research may be compromised, possibly contributing to the inconsistencies within the literature.

Additionally, previous research has focussed on the delivery of blood to the tissue (i.e., CBF), overlooking whether exercise training impacts cerebral oxygen extraction fraction (OEF). OEF is generally inversely proportional to CBF (McFadden et al., 2025; Peng et al., 2014), thus explaining normal age-related OEF increases (Lu et al., 2011; Peng et al., 2014). OEF increases have also been associated with vascular risk progression and white matter hyperintensities (Lin et al., 2023). Importantly, changes in CBF and/or OEF will affect cerebral metabolic rate of oxygen (Fick, 1870). Theoretically, changes in OEF could dictate cerebral tissue function and therefore cognitive functions, potentially contributing to training-induced cognitive benefits.

Therefore, the primary aim of this pre-registered cross-sectional study was to use multiple-delay ASL to accurately compare cerebral haemodynamics in grey matter at rest (i.e., CBF and ATT) between healthy, untrained or long-term endurance trained older adults. Differences in cognitive functions were also analysed to assess any importance of cerebral haemodynamics for cognition. Furthermore, exploratory work examined the potential interplay between cerebral oxygen extraction, cardiorespiratory fitness, and cerebral haemodynamics.

## 2 Methodology

### 2.1 Study design

The data for this publication were collected as part of a larger preregistered study, the FAB Project (https://osf.io/6fqg7). For the present sub-study, transparent changes were made to the original preregistration (https://osf.io/4wdrs) and the materials and data can be found here: https://osf.io/d7aw2/. The data in the present sub-study are from a sub-group of the larger participant cohort, with unique outcome measures to other publications related to the larger project (Fernandes et al., 2024; Feron, Rahman, et al., 2024; Feron, Segaert, et al., 2024; Fosstveit et al., 2024, 2026; Markiewicz et al., 2025; Munns et al., 2026; Prystauka et al., 2025; Rahman et al., 2025), and address *a priori* questions (as per preregistration). The study was approved by the STEM Ethical Review Committee at the University of Birmingham (ERN_20-1107 and ERN_2141). Before enrolment, participants received an information sheet and provided informed consent.

Participants were healthy older adults (aged 60–81 years) that self-reported either to completing <150 minutes/week of moderate-intensity exercise (i.e., untrained group) or to completing regular endurance training for at least the previous 10 years (i.e., trained group). Ninety-four untrained and 31 trained participants were recruited between 10/2021 to 10/2022 and 10/2024 to 01/2025, respectively. The later recruited trained participants were retrospectively pair-matched with untrained participants from the larger existing sample based on sex, age, body mass index (BMI), and education level (listed in order of importance).

All participants were screened for eligibility before completing experimental sessions that involved computerised cognitive tasks, a cardiorespiratory fitness test, and an MRI scan session. Trained participants had additional measurements of haematocrit (venous blood sample), and cerebral oxygen extraction fraction (OEF) as part of MRI session. For the present sample, all outcome measures were completed within 5.4 ± 3.0 weeks, with 2.0 ± 2.5 weeks between cardiorespiratory fitness and MRI sessions. Participants were asked to refrain from vigorous intensity physical activity and caffeine consumption for 24 and two hours prior to the MRI scan, respectively, because these are known to acutely affect cerebral haemodynamics (Clement et al., 2018).

### 2.2 Participants

All participants were community dwelling, cognitively normal, and free from current or historic diagnosis of serious disease. Of the 31 trained participants recruited, *n* = 2 were missing MRI data and *n* = 1 was excluded for an MRI-related incidental finding, leaving *n* = 28 to be pair-matched with participants from the untrained sample. Of the 94 untrained participants from the parent study, *n* = 77 had useable ASL data and were therefore available to be pair-matched with. See Table 1 for participant characteristics of the two pair-matched groups and Section 1 of the Supplementary Material for detailed inclusion criteria and screening procedures.

**Table 1.** Participant characteristics.

	Untrained	Trained
*n* (male:female)	28 (15:13)	28 (15:13)
Age (years)	66.0 ± 5.3	66.2 ± 5.2
BMI (kg/m^2^)	24.9 ± 2.4	23.4 ± 3.2
Highest educational attainment (*n*)		
Compulsory	4	1
Further	12	7
Undergraduate	6	7
Postgraduate	6	13
V̇O_2peak_ (mL/kg/min)	28.4 ± 4.7	42.3 ± 7.2

Values are mean ± standard deviation. BMI: body mass index, V̇O_2peak_: peak oxygen consumption.

Endurance-trained participants self-reported to identify as runners (*n* = 9), cyclists (*n* = 17), or triathletes (*n* = 2) and had participated with their activity for 24 ± 13 years (range = 10–50 years) Over the previous year, endurance activities were engaged with 7 ± 3 hours per week and the average weekly distance completed by cyclists and runners was 127 ± 47 km or 40 ± 10 km, respectively. Regular engagement with competitions was reported by 61% of trained participants.

### 2.3 Outcome measures

#### 2.3.1 Cardiorespiratory fitness

Untrained and trained participants completed different incremental treadmill protocols because the original protocol for untrained participants contained a built-in, and relatively lengthy, lactate threshold protocol which was not required for the latterly recruited trained participants. Untrained participants completed 4 minutes stages with a 1 minute rest period between each stage. Treadmill speed started and remained at 3.8 km/h until either all possible elevation stages were completed (4, 7, 10, 13, 16, 19, and 20% gradient) or individual lactate threshold was reached (2.1 mmol/L rise from first stage). If all elevation stages were completed, 4 minutes stages continued with speed increasing 0.5 km/h per stage until lactate threshold. After reaching lactate threshold, 1 minute stages were completed where speed increased 0.5 km/h per stage (rest periods were removed). Trained participants completed 3 minutes stages with no rest periods. Treadmill speed was adjusted during the warm-up to elicit a heart rate of 100–110 bpm. The first stage began at the pre-determined speed and at 0% gradient, which then increased by 2% each stage. Treadmill test duration for untrained and trained groups were 24.6 ± 6.5 minutes and 24.7 ± 4.2 minutes, respectively.

Respiratory gases (V̇O_2_: oxygen consumption, V̇CO_2_: carbon dioxide production) were recorded continuously using a facemask (7450 V2, Hans Rudolph, USA) and metabolic cart (Untrained: JAEGER Vyntus CPX, Vyaire, USA; Trained: Quark CPET, COSMED, Italy), as was heart rate and rhythm using a 12-lead ECG and ratings of perceived exertion (RPE) (Borg, 1982). All participants were asked to exercise to volitional exhaustion. Cardiorespiratory fitness was determined as peak oxygen consumption (V̇O_2peak_) (i.e., mean of the two highest 30 second intervals). Peak values for untrained and trained participants, respectively, were: percentage of age-predicted peak heart rate (220–age): 104 ± 8% and 108 ± 9%, RER: 1.1 ± 0.2 and 1.0 ± 0.1, RPE: 18.0 ± 1.2 and 18.1 ± 1.0. Discontinuous and continuous incremental protocols to volitional exhaustion elicit similar peak oxygen consumption values (Duncan et al., 1997; Midgley et al., 2007).

#### 2.3.2 MRI outcome measures

An MRI scan session included T1-weighted structural (Magnetization-Prepared Rapid Gradient-Echo [MPRAGE]), functional (echo planar imaging [EPI], untrained only), arterial spin labelling (ASL), and T2-Relaxation-Under-Spin-Tagging (TRUST) (trained only) sequences using a 3-T MRI system (MAGNETOM Prisma, Siemens, Germany) with 32-channel receiver head coil. The focus of this study was the ASL and TRUST data and related scans, analysis of other data acquired can be found elsewhere (Prystauka et al., 2025; Rahman et al., 2025). All untrained participants completed a T1 (5 minutes) sequence and 4 × 15 minutes blocks of an fMRI word-finding task (Rahman et al., 2025) prior to ASL acquisition. All trained participants completed a T1 (5 minutes) and TRUST (1.5 minutes) sequence prior to ASL acquisition. The differences in scanning protocol between untrained and trained participants meant that the ASL sequence began after ~10 and ~65 minutes of lying in the scanner, respectively.

##### 2.3.2.1 Perfusion

Global and regional cerebral blood flow (CBF) and arterial transit time (ATT) in grey matter were estimated using data from a pseudo-continuous ASL sequence with 3D GRASE readout (17.5 minutes) (Kilroy et al., 2014; Wang et al., 2013), see also Acknowledgements. Participants were advised to remain awake during the scan but could choose to keep their eyes open or closed, although this was not formally monitored or recorded.

ASL imaging parameters were set on the console when using the VE11C_GRASE_pCASL_Multidelay_package C2P as follows: repetition time (TR) = 4100 ms, echo time (TE) = 30.56 ms, in-plane resolution = 3.5 mm^2^, slice thickness = 3.5 mm, transversal slices = 32, field of view (FOV) = 224 × 224 mm, background suppression = yes, and post-labelling delays (PLD) = 200, 975, 1425, 1850, 2025, 2150, 2250, and 2300 ms. Four and twelve volumes of data were acquired for PLD of 200–2250 ms and 2300 ms, respectively. The labelling duration was 1.5 seconds and there were two hyperbolic secant inversion pulses during the PLD for background suppression with optimised timing calculated (Shao et al., 2018). PLD times and number of volumes acquired were optimised according to recommendations (Woods et al., 2019). Slices were positioned axially from the motor cortex and angled anterior-posterior in line with the participant’s anterior-posterior commissure (ACPC). A calibration M0 scan was acquired using these same parameters with PCASL and background suppression disabled. The T1-weighted structural scan was acquired to facilitate data analysis, including normalisation to a standard template brain and differentiation of grey and white matter. Structural T1-weighted (MPRAGE) imaging parameters were: TI = 880 ms, TE = 2.03 ms, TR = 2000 ms, voxel size = 1 mm^3^, sagittal slices = 208, FOV = 256 256 × 208 mm, and flip angle = 8°.

Perfusion data were processed using the Oxford ASL toolbox (https://oxasl.readthedocs.io/en/latest/), which uses the FSL FABBER ASL package (FSL v.6.0.7.13) and Bayesian Inference to invert the kinetic model for ASL MRI (BASIL) to compute CBF and ATT maps (Chappell et al., 2009; Groves et al., 2009; Woolrich et al., 2006). Parameters input to the kinetic models to estimate CBF and ATT were: bolus duration = 1.5088 seconds, tissue T1 = 1.3 seconds, arterial blood T1 = 1.65 seconds, and labelling efficiency = 0.85. All other input parameters were kept with default settings appropriate to PCASL acquisition. Partial volume error correction (which implicitly includes adaptive spatial smoothing) of the perfusion maps was performed using default settings in oxford_asl (Chappell et al., 2011; Groves et al., 2009).

Native space average difference maps at each PLD for each participant were visually inspected to ensure data quality. Particular attention was paid to ensure there were no: (1) excessive motion resulting in spurious edge effects in difference maps; (2) brain territories which did not appear to be perfused, due to suboptimal label positioning or unaccounted for vasculature; and (3) focal areas of high intensity in final CBF maps which would have suggested that the PLDs were insufficient. All ASL data used in analyses were deemed to be of good quality (visual inspection).

Global and regional analyses were performed in native (individual participant) and MNI space, respectively. All CBF and ATT values refer to grey matter after partial volume correction. Global CBF and ATT values were calculated from the individual participant’s grey matter masks in native space (see Section 2 of the Supplementary Material). Primary regional analyses included three major vascular territories, fed by either the anterior, middle, or posterior cerebral arteries (ACA, MCA, and PCA, respectively) (Liu et al., 2023) (Supplementary Fig. S2). These primary regional analyses focussed on major vascular territories because we hypothesise that effects of long-term training and cardiorespiratory fitness on cerebral haemodynamics should be relatively homogenous across the brain, whilst enabling investigation of possible anterior-posterior ageing differences (Davis et al., 2008; Meng et al., 2025). Secondary regional analyses included six smaller functional, lobular regions of interest—the cingulate gyrus and frontal, parietal, temporal, occipital, and motor cortices (Supplementary Fig. S4). Secondary regional analyses were performed to enable comparison between previous publications using identical regions of interest (Burley et al., 2021; Feron, Rahman, et al., 2024; Feron, Segaert, et al., 2024; Thomas et al., 2013). Section 2 of the Supplementary Material contains additional information regarding data quality assessments (Supplementary Fig. S1) and configurations of grey matter (Supplementary Fig. S3) and regional masks.

##### 2.3.2.2 Global cerebral oxygen extraction fraction (OEF)

In trained participants only, the T_2_ of blood was non-invasively measured in the superior sagittal sinus (SSS) using the T_2_-relaxation-under-spin-tagging (TRUST) MRI sequence (Jiang et al., 2018, 2021; Lu & Ge, 2008). The single imaging slice was positioned transversely, parallel to the ACPC, 20 mm above the sinus confluence. Imaging parameters for using the TRUST_VE11C_Prisma-XR_R1 sequence were set to: FOV = 220 × 220 × 5 mm^3^, voxel size = 3.44 × 3.44 × 5 mm^3^, TR = 3 seconds, TE = 3.8 ms, TI = 1.02 seconds, labelling slab thickness = 100 mm, gap = 22.5 mm, and scan duration = 1.2 minutes. The T_2_-preparation durations (effective TEs [eTEs]) used were: 0, 40, 80, and 160 ms; with three image pairs collected at each eTE.

A semi-automated TRUST analysis pipeline (Gou et al., 2024; Jiang et al., 2018, 2021; Lu & Ge, 2008) was used. Within this pipeline, data were motion corrected and difference images were computed from pairwise subtraction of control and tag images (Supplementary Fig. S5A). Difference images were visually inspected for motion corruption—no data were excluded for motion corruption. The four voxels within the SSS with the greatest signal intensity were found and spatially averaged (Supplementary Fig. S5B). The change in signal for each eTE was then fitted using Equation 1 to obtain a T_2_ estimate. Delta-R_2_, the 95% confidence interval of the estimated 1/T_2_, represents the uncertainty of experimental measurement. Delta-R_2_ > 5 Hz is suggested as an exclusion criterion for low reliability data (Lin et al., 2023). For the present data, all delta-R_2_ ≤ 3.16 Hz.

$$\Delta S\left(eTE\right)=S_{0}\;\cdot e^{eTE\cdot \left(1/T_{1b}-1/T_{2b}\right)\;}$$ (1)

Individual participant T_2_ estimate was combined with measured haematocrit (Hct) to estimate global cerebral venous oxygenation (Y_v_) using previously derived relationships (Lu et al., 2012). Hct was determined using an automated blood gas analyser (ABL90 FLEX, Radiometer, Ireland) from a venous blood sample (antecubital vein). Arterial blood oxygenation (Y_a_) was estimated for each participant based on age and sex using Equation 2 (Lu et al., 2011).

$$\begin{array}{l} Y_{a}=\;\left(99.77-0.036\times age\right)-\left(1.235\times sex\right)\\ \;\;+\left(0.021\times age\times sex\right) \end{array}$$ (2)

Global cerebral OEF was then determined as a percentage using: (Y_a_–Y_v_) / Y_a_ × 100. OEF data were missing for *n* = 2, leaving a sample of *n* = 26 for analyses. Of these 26, haematocrit data were missing for *n* = 1 and so TRUST analyses used an age- and sex-specific normative value for this participant (Mahlknecht & Kaiser, 2010).

#### 2.3.3 Cognitive functions

The following cognitive tasks were completed on a different day to the exercise and MRI sessions. Untrained participants completed the tasks within a dedicated cognitive testing session as part of a larger project. Trained participants completed the cognitive tasks at the end of the in-person health screening session.

##### 2.3.3.1 Working memory

In a 2-back task based on known concepts (Jaeggi et al., 2008; Kirchner, 1958), participants were presented a 3 × 3 grid. The stimulus was a single white square that continuously appears, disappears, and then reappears in one of the grid squares at random (*n* = 60 trials, 1 second each). Participants identified when the white square appeared in the same location as it did two trials prior (*n* = 12 trials). Trials were excluded from analysis if incorrect or if response time <200 ms or greater than two standard deviations above/below the mean per participant. The primary outcome measure was *d* prime (*d*’), a measure of discriminability, a greater *d*’ indicates superior performance.

##### 2.3.3.2 Attentional Network Task (ANT)

The ANT assessed orienting, alerting, and executive control (J. Fan et al., 2002). The ANT has been shown to have high reliability, and construct and criterion validity in older adults (Ishigami et al., 2016; Macleod et al., 2010). A visual description of the task is shown in Supplementary Figure S18. The stimulus is a row of five arrows, each pointing left or right. As fast and as accurately as possible, participants reported the direction of the centre arrow using the left and right arrow keys. A central fixation cross is displayed for 400 ms, then a fixation cross (500 ms) and cue (100 ms) are presented simultaneously, and then only the fixation cross is displayed for a further 400 ms. A stimulus is then shown for a maximum of 1700 ms. The centre arrow can be congruent or incongruent (i.e., pointing in the same or opposite direction as the flankers, respectively; *n* = 96 each), or neutral (i.e., central arrow flanked by target-irrelevant black blocks, *n* = 96). The stimulus can appear above or below the fixation cross, cued by a black square (*n* = 216) or not cued (*n* = 72). There are three cue conditions: a spatial cue, a centre cue, or a double cue (*n* = 72 each). The spatial cue indicates if the stimulus appears above or below the fixation cross, whereas the stimulus location remains ambiguous for the centre and the double cue. Twelve practice trials are followed by three blocks of 96 trials.

Trials were excluded from analysis if incorrect or if response time <200 ms or greater than two standard deviations above/below the mean per participant. Alerting scores were calculated as the no cue minus the double cue; Orienting scores by centre cue minus the spatial cue; Executive control scores were calculated by the incongruent target minus the congruent target (all for correct responses). High condition difference scores for alerting (no cue–double cue) and orienting (centre cue–spatial cue), and low condition difference scores for executive control (incongruent–congruent), indicate better performance.

##### 2.3.3.3 Processing speed

In a letter comparison task (T. A. Salthouse, 1996), participants were simultaneously presented with two strings of letters at the top and bottom of the screen, for a maximum of 2500 ms after presentation of a fixation cross (1000 ms). Strings were three or six characters long (*n* = 48 trials, 24 each). As fast and as accurately as possible, participants identified whether the strings were the same or different. Mean response time and accuracy were calculated for each participant using data from trials involving only six-character strings. Trials were excluded from analysis if incorrect or if response time <200 ms or greater than two standard deviations above/below the mean per participant.

##### 2.3.3.4 Composite cognitive function

A composite score from the six cognitive function scores above was calculated by summing the Z-scores for each outcome measure (processing speed reaction time and executive control scores were reversed so that all scores were proportional with performance).

### 2.4 Statistical analyses

Unpaired estimation statistics (Ho et al., 2019) assessed between-group differences in V̇O_2peak_, global and regional cerebral haemodynamics at rest (CBF and ATT), and cognitive functions. Differences were calculated as: trained – untrained. Mean differences and Hedges’ *g* with bootstrapped, bias-corrected 95% confidence intervals are reported, as well as *P* values from two-sided permutation tests. The Bonferroni correction adjusted *P* values for multiple comparisons (*P*_adjusted_). Levenes’ tests assessed homogeneity of variance in CBF and ATT between groups. Groups were then collapsed (*n* = 56) to investigate broader associations using multiple linear regressions (bootstrapped with 5000 samples, bias-corrected 95% confidence intervals) (SPSS Statistics v.29, IBM, USA), including between cerebral haemodynamics and V̇O_2peak_ (CBF *or* ATT ~ age + sex + BMI + V̇O_2peak_) or cognitive functions (composite cognitive score ~ age + sex + education + CBF *or* ATT). Cognitive data were incomplete for *n* = 1 trained participant, leaving *n* = 55 for regressions including cognitive data. Exploratory OEF analyses were restricted to trained participants only (*n* = 26) using a multiple linear regression (OEF ~ age + sex + BMI + V̇O_2peak_ + CBF *or* ATT).

## 3 Results

### 3.1 Between-group differences

By design, V̇O_2peak_ was greater in trained participants (13.9 [10.8, 17.1] mL/kg/min, *P* < 0.001, Hedges’ *g* = 2.26 [1.53, 2.85]) (Table 1; Supplementary Fig. S6).

Clear mean differences in global grey matter CBF were not apparent (Fig. 1A: 5.6 [-2.6, 13.9] mL/100 g/min, *P* = 0.197, Hedges’ *g* = 0.34 [-0.19, 0.88]). However, trained participants had shorter grey matter ATT globally (Fig. 1B: -0.11 [-0.21, -0.02] seconds, *P* = 0.031, Hedges’ *g* = 0.58 [0.04, 1.11]). Descriptive means can be found in Supplementary Table S1. Results were similar after controlling for educational attainment (see Section 3 of the Supplementary Material).

**Fig. 1.**
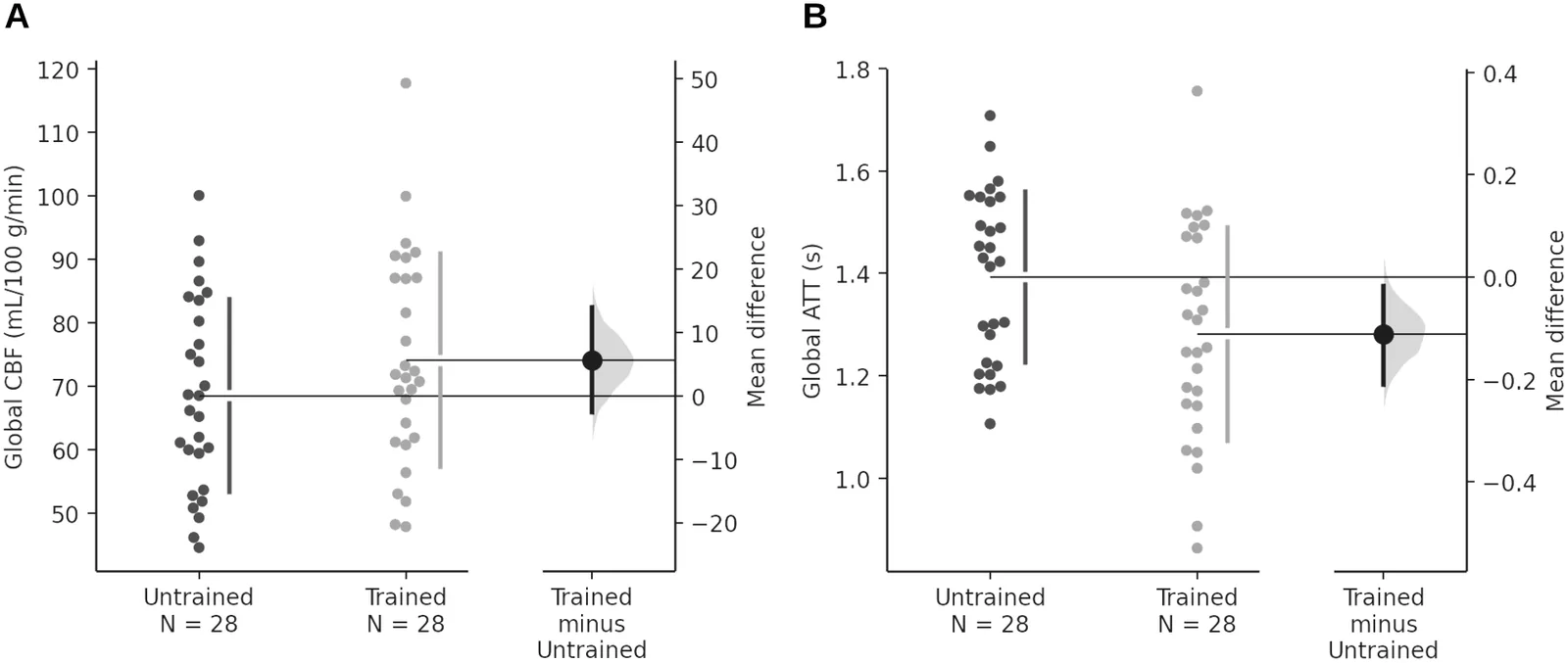
Individual participant data for each group including group mean and standard deviation, and the mean between-group difference with 95% confidence intervals in (A) global cerebral blood flow (CBF) and (B) arterial transit time (ATT) in grey matter at rest between untrained and trained older adults.

Global between-group difference maps for CBF and ATT were made to visualise any regional patterns, data are shown in Figure 2. Reinforcing the global quantitative analyses, the relative shortening in ATT of trained participants appears relatively homogenous across the brain (Fig. 2B). In contrast, between-group CBF differences are more heterogeneous (Fig. 2A). Most regions do demonstrate small CBF elevations within trained compared to untrained participants, particularly in superior-frontal regions. However, a posterior region appears to show the opposite effect.

**Fig. 2.**
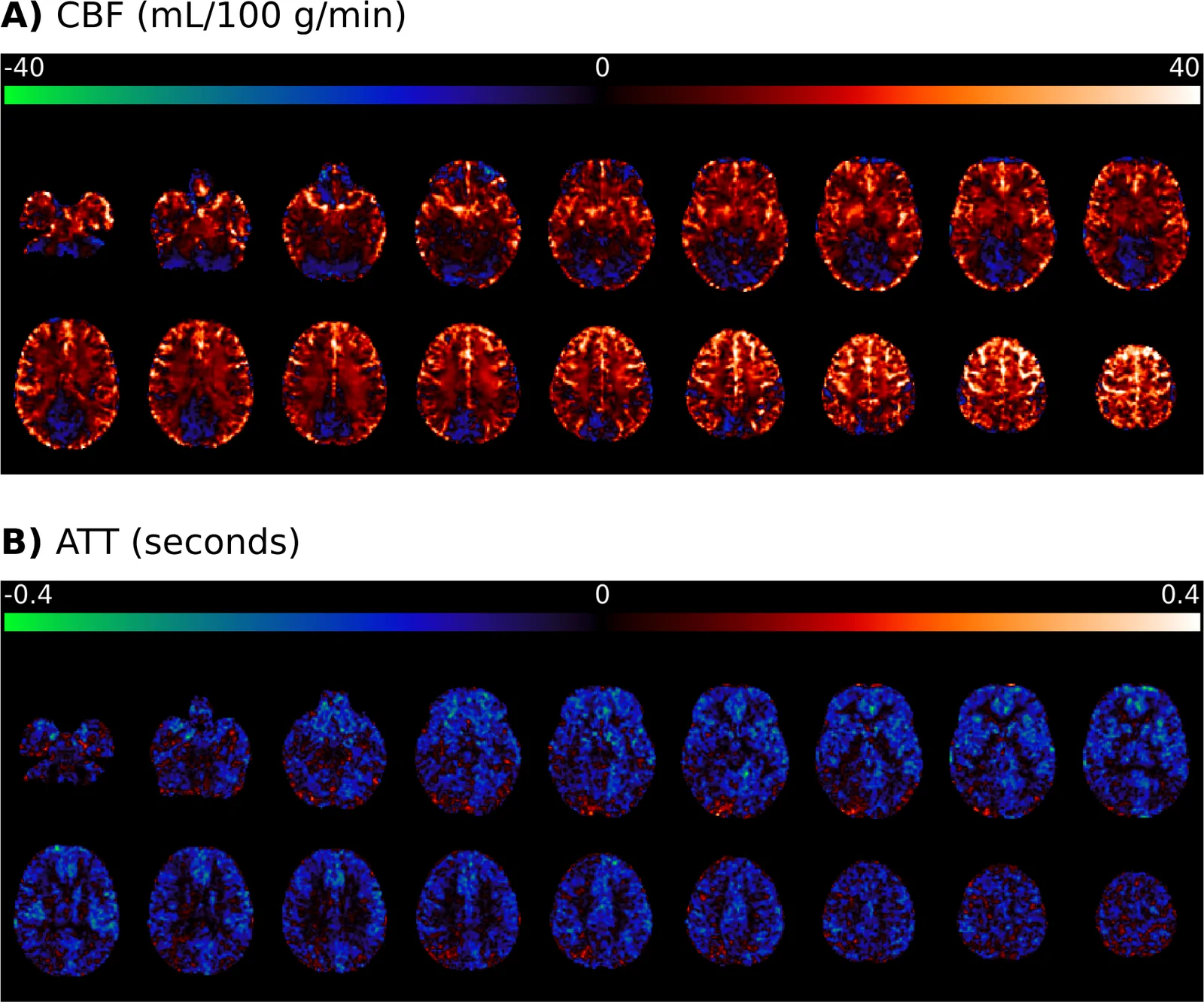
Descriptive brain maps of between-group differences in grey matter (after partial volume correction) (A) cerebral blood flow (CBF) and (B) arterial transit time (ATT). Calculated as: trained – untrained (*n* = 28 per group). Voxels with ≥80% participant data in each group are shown. See also Supplementary Figures S8 (liberal statistical threshold) and S9 (non-partial volume corrected data).

Primary regional analyses of grey matter CBF within the three major vascular territories identified significantly greater CBF in the ACA territory for the trained group (Fig. 3A: 15.8 [3.2, 29.1] mL/100 g/min, *P* = 0.021, Hedges’ *g* = 0.62 [0.09, 1.13]), although this result did not survive adjustment for multiple comparisons (*P*_adjusted_ = 0.064). Significant between-group differences were not evident for CBF in the MCA territory (Fig. 3B: 10.4 [-0.2, 21.9] mL/100 g/min, *P* = 0.071, Hedges’ *g* = 0.48 [-0.05, 1.0]) or the PCA territory (Fig. 3C: 0.1 [-8.6, 8.2] mL/100 g/min, *P* = 0.984, Hedges’ *g* = 0.00 [-0.52, 0.55]). See Supplementary Table S1 for descriptive means and Supplementary Figure S7 for distributions. There were no between-group differences in CBF variance.

**Fig. 3.**
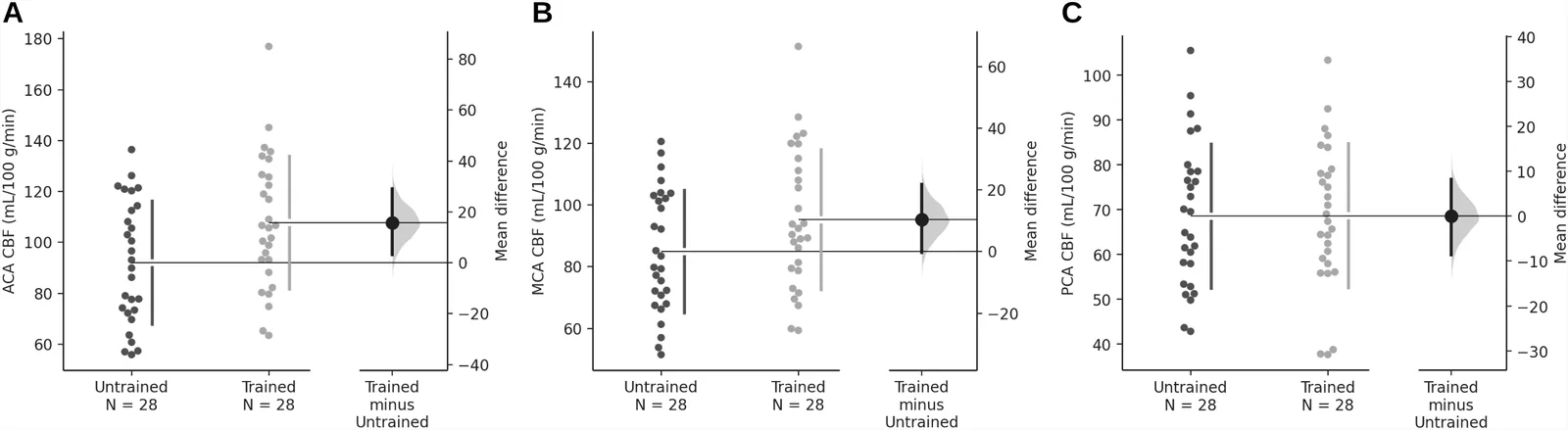
Individual participant data for each group including group mean and standard deviation, and the mean between-group difference with 95% confidence intervals in grey matter cerebral blood flow (CBF) within regional vascular territories at rest between untrained and trained older adults. (A) Anterior cerebral arteries (ACA) territory, (B) middle cerebral arteries (MCA) territory, and (C) posterior cerebral arteries (PCA) territory. Supplementary Figure S2 shows these regions.

There were no significant differences in grey matter ATT within the ACA territory (Fig. 4A: -0.08 [-0.17, 0.00] seconds, *P* = 0.084, Hedges’ *g* = -0.46 [-0.99, 0.06]), MCA territory (Fig. 4B: -0.10 [-0.19, -0.01] seconds, *P* = 0.053, Hedges’ *g* = -0.53 [-1.06, 0.01]), or PCA territory (Fig. 4C: -0.07 [-0.16, 0.02] seconds, *P* = 0.153, Hedges’ *g* = -0.39 [-0.92, 0.16]) vascular territories. See Supplementary Table S1 for descriptive means and Supplementary Figure S7 for distributions. There were no between-group differences in ATT variance.

**Fig. 4.**
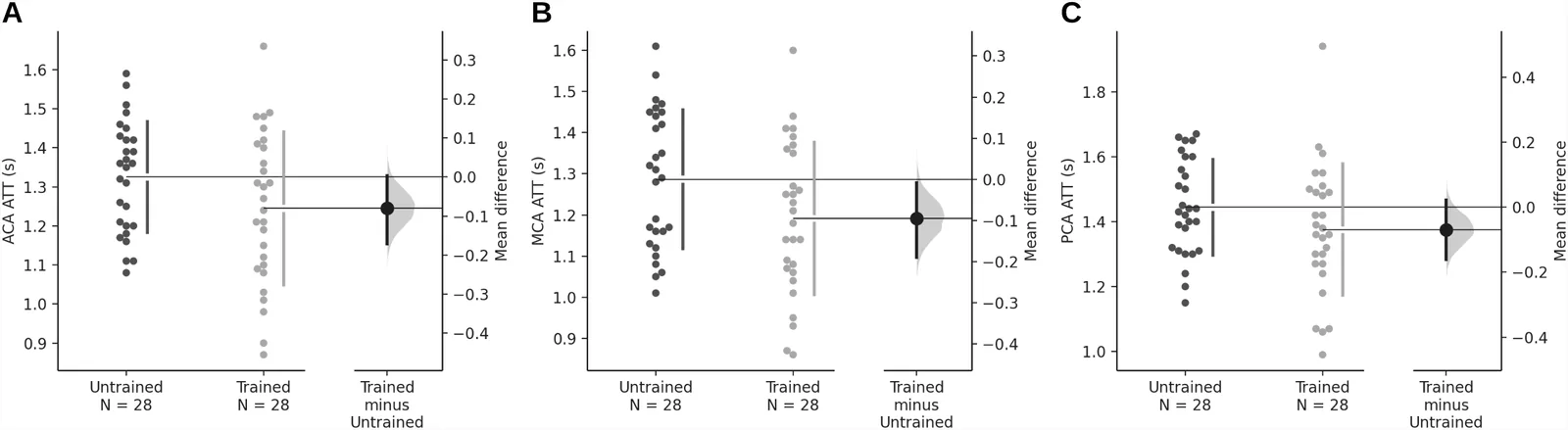
Individual participant data for each group including group mean and standard deviation, and the mean between-group difference with 95% confidence intervals in grey matter arterial transit time (ATT) within regional vascular territories at rest between untrained and trained older adults. (A) Anterior cerebral arteries (ACA) territory, (B) middle cerebral arteries (MCA) territory, and (C) posterior cerebral arteries (PCA) territory. Supplementary Figure S2 shows these regions.

White matter CBF and ATT data within ACA, MCA, and PCA territories were also available and are presented in Supplementary Figures S10 and S11, respectively. White matter broadly demonstrates the same patterns as the grey matter data.

Secondary analyses of six lobular, functional regions are shown in Section 3 of the Supplementary Material (Supplementary Figs. S12 and S13). Briefly, no between-group differences were significant. (Supplementary Table S2).

Regarding cognitive functions, there were no statistical between-group differences in composite cognitive scores or any of the individual cognitive measures (Table 2; Supplementary Figs. S15 and S16).

**Table 2.** Between-group differences in cognitive functions.

Cognitive function	Mean difference	Hedges’ *g*
Composite (Z-score)	-0.05 [-1.72, 1.74]	-0.01 [-0.59, 0.50]
*Processing speed*		
Accuracy (%)	-1.9 [-7.6, 4.5]	-0.16 [-0.73, 0.38]
Reaction time (ms)	-7 [-20, 6]	-0.29 [-0.8, 0.27]
*Working memory*		
2-back *d’*	-0.01 [-0.49, 0.43]	-0.01 [-0.55, 0.50]
*Attentional network task (ms)*
Alerting	3 [-6, 13]	0.17 [-0.37, 0.68]
Orienting	-4 [-16, 7]	-0.18 [-0.68, 0.34]
Executive control	11 [-15, 40]	0.21 [-0.34, 0.72]

Mean differences calculated as: trained – untrained. Data include [95% confidence intervals].

### 3.2 Collapsed sample associations

Multiple linear regressions found no association between V̇O_2peak_ and global CBF (β = 0.12 [-0.28, 0.52], *P* = 0.538) or V̇O_2peak_ and global ATT (β = -0.25 [-0.59, 0.07], *P* = 0.157). In both global models, age predicted lower CBF (β = -0.32 [-0.54, -0.12], *P* = 0.007) and longer ATT (β = 0.37 [0.10, 0.57], *P* = 0.002). Regional analyses also found no association between V̇O_2peak_ and CBF (for all, *P* > 0.24) or ATT (for all, *P* > 0.25).

Composite cognitive scores were not associated with global or regional CBF (for all, *P* > 0.22) or ATT (for all, *P* > 0.23).

Figure 5 shows unadjusted bivariate associations between age and global CBF and global ATT split by training group.

**Fig. 5.**
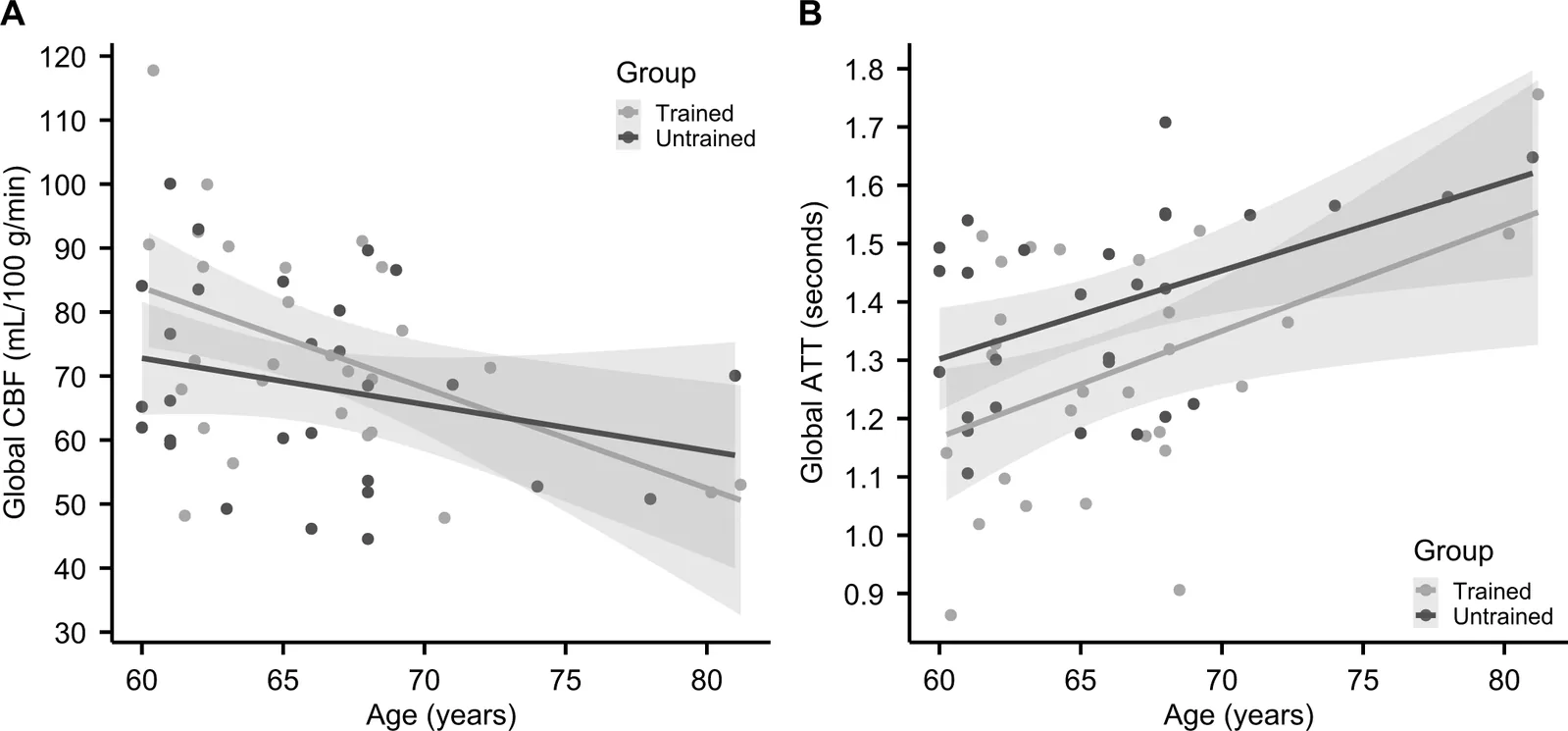
Bivariate unadjusted associations between age and (A) global cerebral blood flow (CBF) or (B) global arterial transit time (ATT) in grey matter at rest for untrained (grey) and trained (dark grey) groups.

### 3.3 Exploratory cerebral oxygen extraction fraction analyses

Mean OEF in trained participants (*n* = 26) was 34.1 ± 6.2% (range = 23.7–46.9%). The multiple linear regression model including CBF found a significant inverse association between OEF and age (β = -0.52 [-0.89, -0.23], *P* = 0.007), and between OEF and global CBF (β = -0.96 [-1.21, -0.75], *P* = 0.002) (Fig. 6A). In contrast, the model including ATT found no significant association between OEF and age (β = -0.25 [-1.16, 0.92], *P* = 0.304), or between OEF and global ATT (β = 0.42 [-0.02, 0.73], *P* = 0.112) (Fig. 6B). A bivariate correlation between OEF and age found no significant association (r = -0.12 [-0.41, 0.29], *P* = 0.606). Neither model found significant associations between OEF and BMI (*P* > 0.53) or V̇O_2peak_ (*P* > 0.74). Simple bivariate correlations between OEF and CBF or ATT are shown in Supplementary Figure S17.

**Fig. 6.**
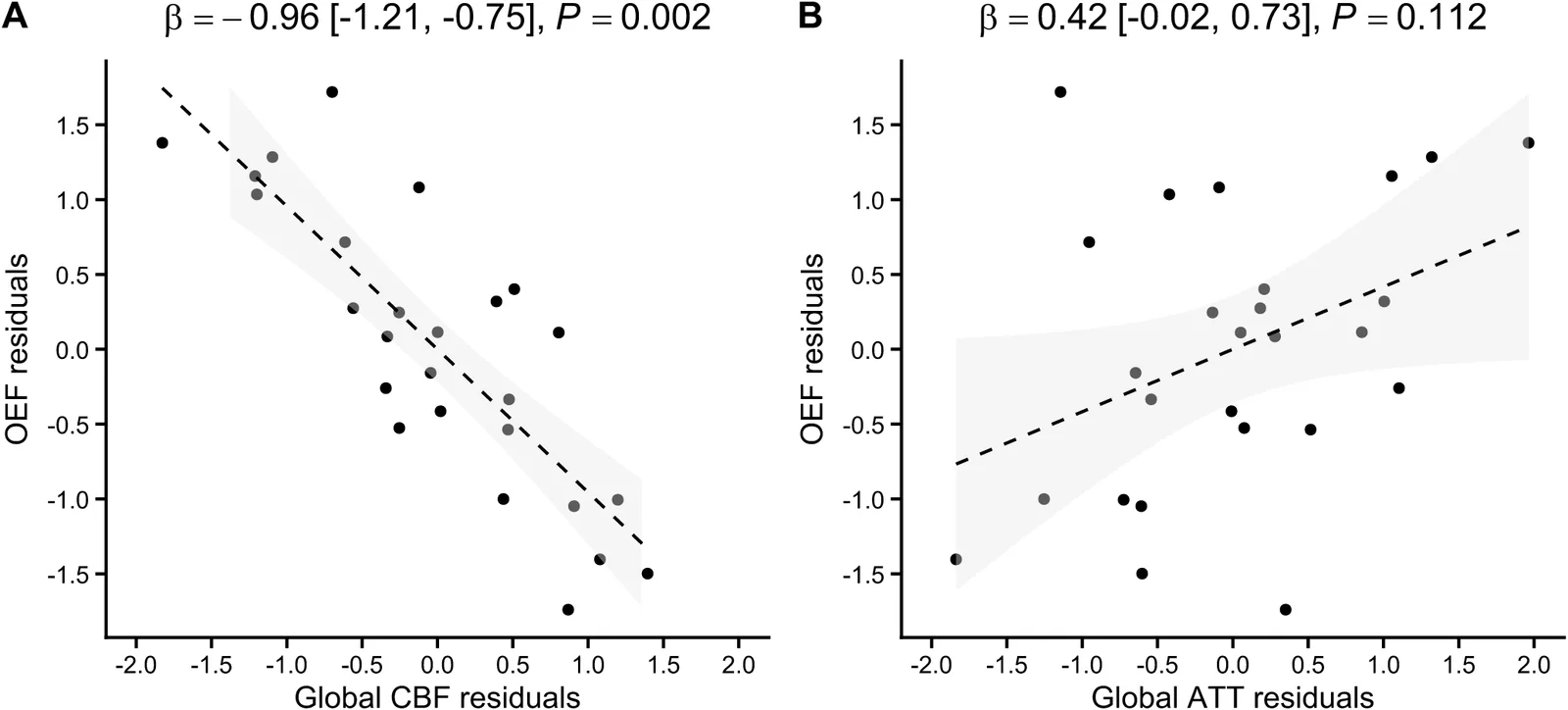
Partial regression associations between global cerebral oxygen extraction fraction (OEF) and (A) global cerebral blood flow (CBF), or (B) global arterial transit time (ATT) in trained participants (*n* = 26). Model: OEF ~ age + sex + BMI + V̇O_2peak_ + CBF *or* ATT. BMI: body mass index, V̇O_2peak_: peak oxygen consumption.

## 4 Discussion

The present study assessed cross-sectional differences in grey matter cerebral haemodynamics at rest between long-term exercise trained older adults (i.e., ≥10 years) and healthy untrained peers. We estimated cerebral haemodynamics using multiple post-labelling delay ASL, which means that our study is the first investigation of the differences from long-term training in ATT and in CBF when not confounded by ATT differences. Comparisons found no clear differences in global CBF, but trained participants had shorter ATT globally (-0.11 [-0.21, -0.02] seconds). Cognitive functions did not differ between the groups, and composite cognitive scores were not associated with cerebral haemodynamics at rest. Global cerebral OEF data collected from trained participants revealed a strong inverse association with global CBF, but no association with cardiorespiratory fitness.

### 4.1 Cerebral blood flow

Similar to previous studies (Burley et al., 2021; Sugawara et al., 2020; Thomas et al., 2013), there was no clear statistical between-group difference in global CBF. However, Figure 2 shows greater CBF within trained participants broadly across the brain, except within a posterior region where CBF was conversely lower. This regional heterogeneity potentially contributed to the null global findings. Our non-significant group difference in global CBF (5.6 [-2.6, 13.9] mL/100 g/min) corresponds to ~18 years of normal age-related changes (Damestani et al., 2023). Furthermore, a medium-to-large, but non-significant (*P_adjusted_* = 0.064), effect was seen for greater CBF specifically within the ACA vascular territory in trained participants. This ACA difference offers some support that long-term training may slow normal age-related CBF declines (Damestani et al., 2023), which were also observed in the present sample. Previous evidence of greater regional CBF in trained participants are limited to only relative posterior cingulate cortex (PCC) CBF from a voxel-wise analysis (Thomas et al., 2013) or absolute occipitoparietal CBF, but not the hippocampus, precuneus, or PCC (Tarumi et al., 2013). Similar to previous studies (Burley et al., 2021; Feron, Segaert, et al., 2024; Flodin et al., 2017), we found no association between individual CBF and cardiorespiratory fitness level within the combined sample. An association could still exist but may vary depending on how long high fitness levels have been sustained for.

Importantly, unlike most previous studies comparing untrained and trained populations, we used multiple post-labelling delay ASL to adjust CBF for individual and regional differences in ATT (Dai et al., 2017; Woods et al., 2024). This improves not only CBF estimation accuracy, but also the validity of group comparisons because the present data indicate that ATT does vary with training status. Burley et al. (2021) also used multiple post-labelling delay ASL but was restricted by a small sample size (*n* = 14). Therefore, we present the first investigation of differences from long-term training and fitness levels in ATT and in CBF when not confounded by ATT differences.

Our group-level difference map (Fig. 2) also shows a posterior region where CBF is lower for trained participants. The PCA territory mask includes most of this region alongside neighbouring regions with increased CBF, potentially contributing to the null between-group difference in PCA CBF. It should also be noted that the PCA territory is fed from the basilar artery whilst the ACA and MCA are fed from the internal carotid arteries (ICA). The ASL sequence will have tagged the blood in the basilar artery and ICA, however the pattern of results presented here is not changed if the ACA and MCA regions are combined in these data (data not shown). Posterior brain regions can experience some of the largest and most variable age-related CBF declines (Chen et al., 2011; Damestani et al., 2023; Meng et al., 2025), and acute blood flow/velocity responses to exercise may differ between posterior and middle cerebral arteries (Sato et al., 2011; Smith et al., 2012; Steventon et al., 2018). Furthermore, posterior circulation anatomical variants could affect cerebral haemodynamics and reactivity (Warnert et al., 2016) and vascular territory boundaries. Future research should consider differential anterior-posterior regional effects of exercise training on CBF and attempt to define vascular territories on an individual level (Paiva et al., 2007) to account for morphological differences.

Several factors could have contributed to the lack of clearer between-group differences in the present data. Firstly, post hoc analysis indicates power is 62% when using the largest regional effect size and an alpha of 0.05 (pwr, RStudio). Secondly, median [interquartile range] global CBF within our untrained group was considerably higher than previously reported for 221 healthy adults aged 60–79 years (67.3 [56.5–81.9] vs. 53.5 [47.1–60.2] mL/100 g/min), also estimated using multiple-delay ASL (Yetim et al., 2023). In addition, objective physical activity measurements as part of the larger project (Fosstveit et al., 2024) revealed 85% of untrained participants actually met activity guidelines they self-reported not to. Therefore, the brain health within the untrained group may not represent the general population, masking possible differences with long-term trained older adults.

Interpreting CBF responses to short- or medium-term exercise interventions in older adults (i.e., ≤6 months) is difficult because training-induced functional vascular adaptations precede structural adaptations, meaning an equilibrium is unlikely to have been reached. The present data align with Tomoto et al. (2023), reporting increased global volumetric CBF after a 1-year intervention, indicating that sustaining exercise training over a longer period may induce broader CBF elevations. Accumulated 10-year physical activity scores also predict greater PCC CBF in older adults (Boraxbekk et al., 2016). Broader CBF changes could result from larger scale structural cerebrovascular adaptations within the arterial tree. For example, long-term trained older adults exhibit a greater number of small cerebral vessels (radius = 0.5–0.6 mm) in the anterior and middle circulations (Bullitt et al., 2009). However, long-term longitudinal analyses are required to confirm these hypotheses.

Taken together, the present data found no statistically significant differences in grey matter CBF at rest between untrained and long-term endurance trained older adults. A medium-to-large effect size of greater CBF for trained participants was evident within the ACA vascular territory which warrants further investigation with longitudinal designs and more heterogeneous samples. Finally, we reinforce the importance of using multiple post-labelling delays within ASL research to adjust for regional, individual, and training status-related ATT variability.

### 4.2 Arterial transit time

The present data indicate that arterial blood takes less time to travel to the cerebral tissue from the arteries within the labelling plane (i.e., internal carotid and basilar) at rest in trained older adults (i.e., shorter global ATT)—likely reflecting a slowing of normal age-related ATT lengthening (Damestani et al., 2023), a relationship also observed in the present sample (Fig. 5). Our group difference in global ATT (-100 [-210, -20] ms) corresponds to ~13 years of normal age-related changes (Damestani et al., 2023). ATT may be more sensitive to age-related changes than CBF (Damestani et al., 2023; Feron, Segaert, et al., 2024), potentially contributing to the clearer between-group difference in ATT.

Only three studies have investigated this topic, two of which are from the present research group that include the parent sample of the present untrained group. From an independent participant sample, Burley et al. (2021) identified no differences in ATT between untrained and trained older adults, but the sample size was small (*n* = 14) (Burley et al., 2021). In contrast, in a partly overlapping dataset, we previously identified that greater cardiorespiratory fitness was associated with prolonged ATT in parietal and occipital regions (Feron, Segaert, et al., 2024). Our previous finding opposes the group difference reported here (i.e., prolonged ATT in untrained participants), but all participants in Feron, Segaert, et al. (2024) were untrained older adults. As expected, given the partly overlapping untrained dataset, a similar trend for a positive association between global ATT and cardiorespiratory fitness was seen in the present untrained group, but no association was present within the trained group (Supplementary Fig. S14). Future research should consider that cardiorespiratory fitness-brain health associations may differ depending on long-term training status. Finally, we previously reported no impact of 6-month exercise training on ATT (Feron, Rahman, et al., 2024). Together, the limited available data indicate that long-term, but not short-term, exercise training may affect ATT, highlighting a potential involvement of structural vascular factors that tend to manifest over a longer period (e.g., vascular remodelling). Furthermore, the relationship between cardiorespiratory fitness and brain vascular health may change with training status, potentially plateauing at higher fitness levels and requiring non-linear approaches to improve understanding.

Theoretically, the key determinants of ATT are the velocity of the blood and/or the path length of which it travels. Indeed, a strong inverse association is evident between cerebral blood velocity and ATT (Burley et al., 2021), and evidence indicates that long-term exercise training increases mean cerebral blood velocity in male samples (Ainslie et al., 2008; Bailey et al., 2013). However, this effect is less clear in mixed-sex samples (Bliss et al., 2023; Burley et al., 2021; Zhu et al., 2013). Exactly how long-term exercise training would elevate cerebral blood velocity warrants investigation, but may be related to mechanisms that reduce resistance to blood flow (e.g., improved endothelial function, arterial elasticity, or wider vessel lumens) (Green et al., 2017). Regarding cerebrovascular structure, training may slow normal age-related changes such as increased whole-brain vessel tortuosity or length of larger cerebral arteries (Mouches et al., 2021; B. Zhang et al., 2022). Indeed, lower cerebral vessel tortuosity is evident in trained older adults (Bullitt et al., 2009). These adaptations would shorten the vascular path length and could reduce blood flow resistance, explaining the shorter ATT we observed.

Further research is required to quantify and understand long-term training-induced structural cerebrovascular changes because these may influence haemodynamics and cerebral tissue function. Moreover, given that ATT appears sensitive to age and long-term exercise training, it’s long-term predictive capacity for cognitive decline, dementia, or stroke incidence should be investigated.

### 4.3 Cognitive functions

Cerebral haemodynamics are hypothesised to be important for exercise training-related benefits to cognitive functions (Bliss et al., 2021; Cabral et al., 2019). The present data cannot be used to support this hypothesis because there was no evidence of cognitive benefits from long-term exercise training, despite some between-group differences in cerebral haemodynamics. Although cognitive benefits of exercise training and higher cardiorespiratory fitness in older adults are commonly reported (Barha et al., 2017; Northey et al., 2018; Oberlin et al., 2025), disparities do exist (Kelly et al., 2014; Young et al., 2015). In the present study, the lack of cognitive differences may be related to the high health status of the untrained group due to extensive health-related inclusion criteria and high objective physical activity levels (discussed previously). Furthermore, although untrained education levels were lower overall, 43% had still completed undergraduate or postgraduate education. Finally, the present sample included primarily the younger-old (86% aged 60–69 years), whereas cognitive functions typically only begin to considerably worsen from approximately 60 years of age (Gorbach et al., 2017; Nyberg et al., 2020; Zaninotto et al., 2018). These factors could have meant that age-related cognitive declines were minimal and that little individual variability was present.

### 4.4 Cerebral oxygen extraction fraction

Previous research investigating the importance of exercise training to brain vascular health in older adults has focussed on the delivery of blood to the tissue (i.e., CBF) rather than how the brain extracts nutrients from the blood (i.e., oxygen and glucose), despite its theoretical importance to cerebral tissue function. OEF is generally considered to increase with age (Lin et al., 2023; Lu et al., 2011; Peng et al., 2014), whereas both increases and decreases have been reported in disease states (e.g., stroke or vascular risks vs. dementia, respectively) (A. P. Fan et al., 2019; Jiang et al., 2020; Lin et al., 2023; Thomas et al., 2017). Therefore, the present study explored novel associations between cerebral OEF and cardiorespiratory fitness or BMI in trained older adults (*n* = 26), reporting no associations in the present trained sample. Our data do not show a robust effect of age on OEF, likely due to the limited age range.

The present data do show a strong inverse association between OEF and global CBF, as has been reported previously (McFadden et al., 2025; Peng et al., 2014). Unlike the present data, McFadden et al. (2025) also reports a positive association between OEF and ATT in older adults; however, unlike our trained group, their sample included varying degrees of vascular risk and cognitive impairment. Cerebral haemodynamics likely have a synergistic, compensatory relationship with OEF to help maintain cerebral metabolic rate and thus maintain cerebral tissue function and/or integrity. However, the compensatory interplay of these metrics (e.g., increased OEF for low CBF or vice versa) and consistency between individuals is not clear. Therefore, future research should endeavour to assess both cerebral haemodynamics and OEF, providing a more comprehensive picture of brain vascular health. Using longitudinal and exercise intervention-based designs will help to understand the interplay between the delivery and utilisation of nutrients to the cerebral tissue in older adults, and whether these mechanisms mediate exercise-related brain health benefits or long-term cognitive outcomes.

### 4.5 Limitations and considerations for future research

The present sample was relatively homogeneous, predominantly consisting of physically and cognitively healthy, educated, white, older adults, aged 60–81 years, with the majority between 60 and 70 years. Untrained participants were objectively more physically active than they self-reported to be. These factors may have impacted on our ability to detect relationships between training status, brain vascular health, and cognitive functions.

Additionally, untrained participant data were collected as part of a larger project which included additional MRI sequences. This meant that untrained ASL measurements were performed after an fMRI task during which they were laying supine for ~65 minutes in comparison to only ~10 minutes of supine rest for trained participants. Cerebral artery blood velocity, a common proxy for CBF (Willie et al., 2011) which is also inversely associated with ATT (Burley et al., 2021), generally increases in responses to cognitive demand (Csipo et al., 2021; Frauenfelder et al., 2004; Sorond et al., 2008). Similarly, compared to seated, 20 minutes of supine rest has been shown to increase CBF and cerebral artery blood velocity (Possnig et al., 2026). These stimulatory haemodynamic effects of cognitive demands and postural changes could have increased CBF and/or shortened ATT in our untrained sample. However, our data show widespread regions of lower CBF and longer ATT in the untrained group.

Furthermore, future research assessing cerebral haemodynamic changes should consider controlling for partial pressure of end-tidal CO_2_ (P_ET_CO_2_), which was not done in the present study. P_ET_CO_2_ is a proxy for arterial partial pressure of CO_2_ (P_a_CO_2_), the most powerful regulator of CBF (Willie et al., 2014), which could be manipulated by anxiety-induced ventilatory changes during an MRI scan. Our trained group included both runners and cyclists. These modes of exercise may induce different adaptive stimuli (e.g., blood velocity and shear-stress) (Furlong et al., 2020) which might have partially masked between-group physiological differences in perfusion.

### 4.6 Conclusion

The present cross-sectional study assessed differences between long-term endurance exercise trained (≥10 years) older adults and untrained peers. There were no clear differences in CBF globally, but global ATT was shorter in trained participants (i.e., blood takes less time to travel to the brain from the carotid arteries)—training may thus counteract age-related ATT lengthening effects. The present data do indicate that long-term training may induce regional CBF increases within anterior vascular territories, although in the present data these were not statistically significant. We demonstrate the importance of using multiple-delay arterial spin labelling within exercise research to adjust CBF estimates for training-status-related ATT differences. Cognitive functions did not differ between groups and were not associated with cerebral haemodynamics. Cerebral OEF estimates in trained participants were not associated with cardiorespiratory fitness but were strongly inversely related to CBF, highlighting that CBF and OEF should not be investigated in isolation. Longitudinal and intervention-based studies with a more heterogenous older adult sample are required to confirm cerebral haemodynamic changes following long-term training and determine implications for cognitive functions.

## Data and Code Availability

The data for the present study were collected as part of a larger study, The FAB Project: preregistration: https://osf.io/6fqg7 and https://osf.io/4wdrs; materials and data: https://osf.io/d7aw2/.

## Supplementary Material

Supplementary Material

## Supplementary Materials

Supplemental material contains additional information regarding Materials and Methods and Results sections. Supplementary material for this article is available here: https://doi.org/10.1162/IMAG.a.1427#supplementary-data.
