# Supplementary material for: Differences in cerebral haemodynamics associated with long-term endurance training in older adults
Source: Imaging Neurosci (Camb). 2026 Nov 4;4:IMAG.a.1427. doi: 10.1162/IMAG.a.1427 (PMC13736791; doi:10.1162/IMAG.a.1427)
Supplement: Supplementary file 1 [file IMAG.a.1427_supp.pdf]

Supplementary Material

Differences in cerebral haemodynamics associated with long-term endurance training in older adults

1) Participant enrolment

Inclusion criteria

Inclusion criteria were: 1) aged 60-85 years; 2) do <150 mins of moderate physical activity per week; 3) monolingual; 4) right-handed; 5) no current/historic diagnosis of cardiovascular, metabolic, respiratory, neurological, kidney, liver, or cancerous disease; 6) resting electrocardiogram (ECG) and blood pressure screened by clinician (i.e., no severe ECG abnormalities (e.g., ST depression, long QT, heart block, wide QRS) and systolic/diastolic blood pressure of <160/<90 mmHg, respectively); 7) Montreal Cognitive Assessment (MoCA) score ≥23; 8) not taking neurotransmitter-altering medication; 9) vaccinated against COVID-19. 9) deemed safe to enter MRI scanner by a qualified MRI-operator; 10) no language impairments; 11) no post-traumatic stress disorder (PTSD); 12) non-smoker of at least 5 years.

Health screening: electrocardiogram (ECG), blood pressure, and cognitive impairment

Participants completed a resting 12-lead ECG (Cardiosoft, Vyaire, USA), three resting blood measurements (705IT, Omron, Japan), and the Montreal Cognitive Assessment (MoCA). Participants were excluded for severe ECG abnormalities, MoCA scores <23 (Carson et al., 2018), and resting systolic/diastolic blood pressure of >160/>90 mmHg, respectively. Excluded participants were referred to their GP.

2) MRI acquisition and analyses

For each participant, ASL data at each PLD (difference of tag and control averaged over repeats) and grey matter masks in native space were visually inspected. Figure S1 shows two examples of ASL data from the larger data set which were excluded after this visual data quality inspection step. Native space grey matter masks were further thresholded at 0.5 probability to ensure only voxels containing primarily grey matter were included in calculations of CBF and ATT. One participant's native space mask contained voxels with incorrect assignment to grey matter, primarily around the eyes and nasal cavity, which were manually removed.

Structural and ASL MRI data were aligned to the MNI standard brain using fsl_anat (https://fsl.fmrib.ox.ac.uk/fsl/fslwiki/fsl_anat) for regional analyses. Registrations to MNI space were visually inspected. Primary regional analyses included masks of three major vascular territories (Figure S2) that are publicly available (Liu et al., 2023). Prior to territory mask application, partial volume corrected CBF and ATT data were masked with a grey matter mask made using the MNI standard tissue priors in FSL to exclude voxels < 42% grey matter probability (Figure S3). Secondary regional analyses included grey matter masks of six functional regions (Figure S4). These six masks were in MNI space and defined using the MNI structural atlas (temporal lobe only) or from the conjunction of the relevant regions from the Harvard atlas in FSL. Masks of these functional regions have been used previously (Burley et al., 2021; Feron et al.,

2024b, 2024a), and masks were broad because there were no specific *a priori* hypotheses of regions that would be affected by exercise training or associated with cognitive functions.

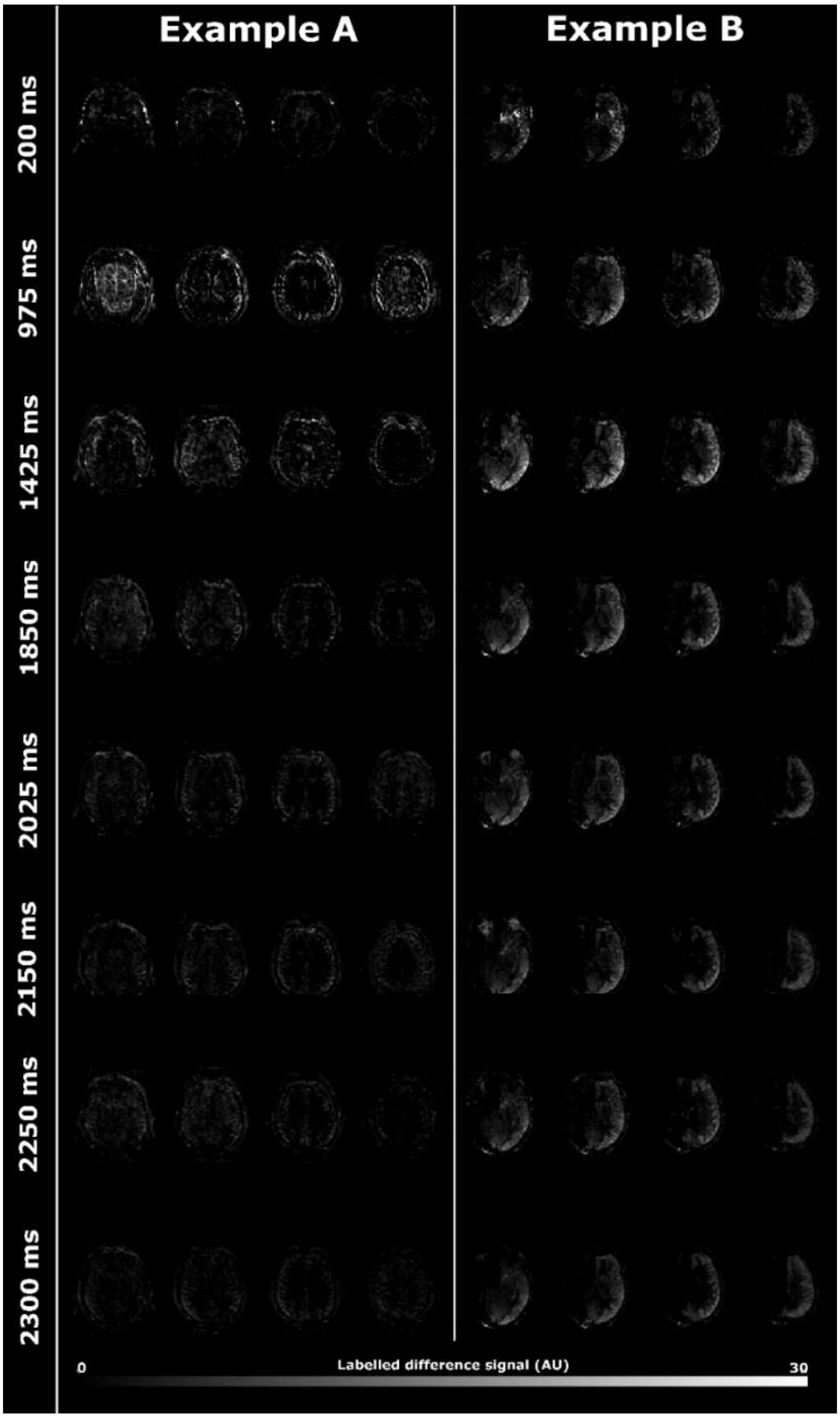

Figure S1: Examples of arterial spin labelling difference maps at each post-labelling delay from two excluded participants. AU: arbitrary units.

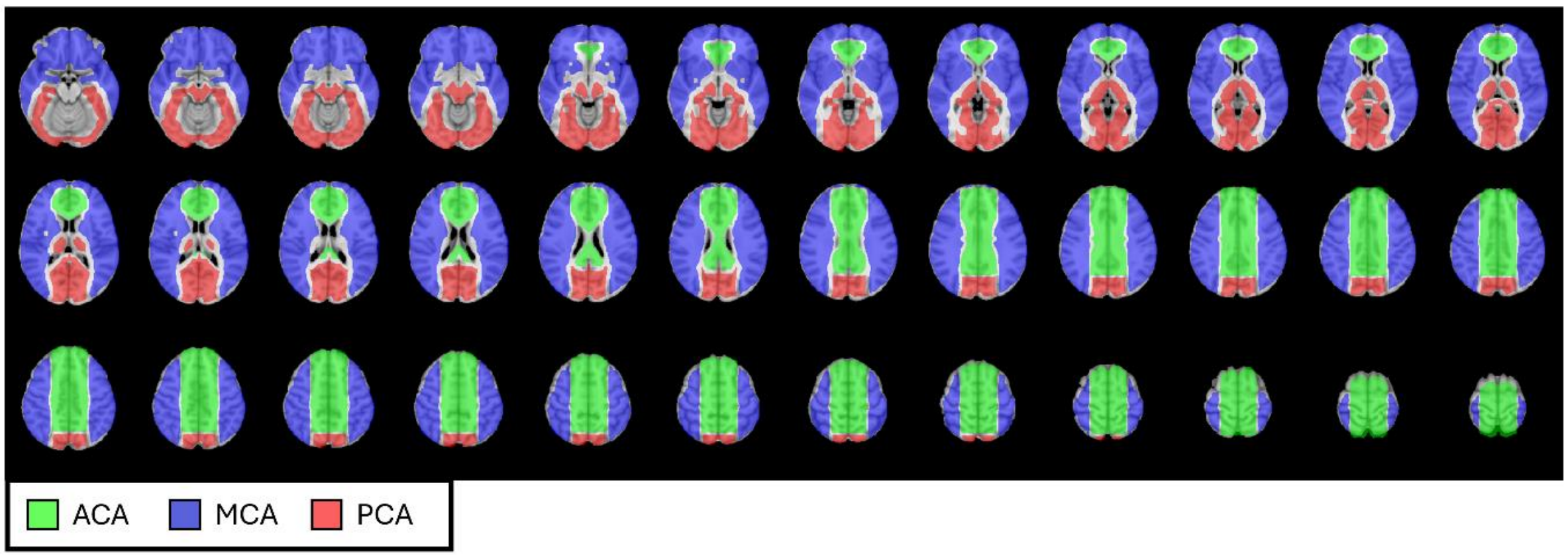

Figure S2: Masks of vascular territories in MNI space used for regional analyses. ACA: anterior cerebral arteries, MCA: middle cerebral arteries, PCA: posterior cerebral arteries.

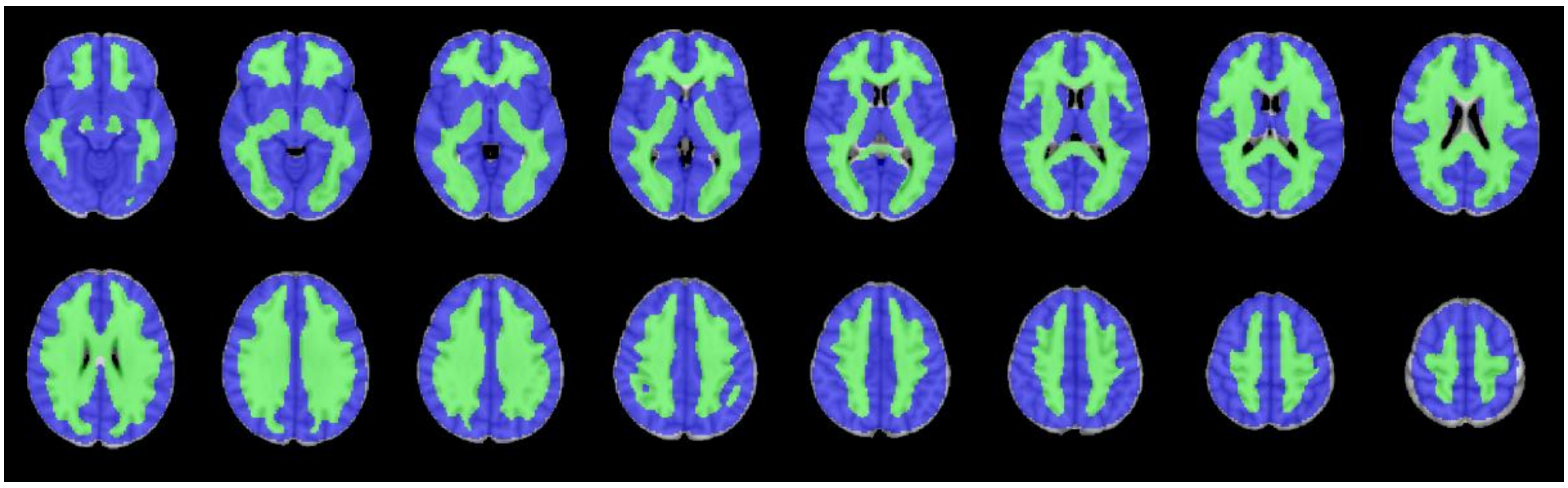

Figure S3: Manually created masks for grey matter (blue) and white matter (green) regions in MNI space using tissue priors for the MNI152 standard template.

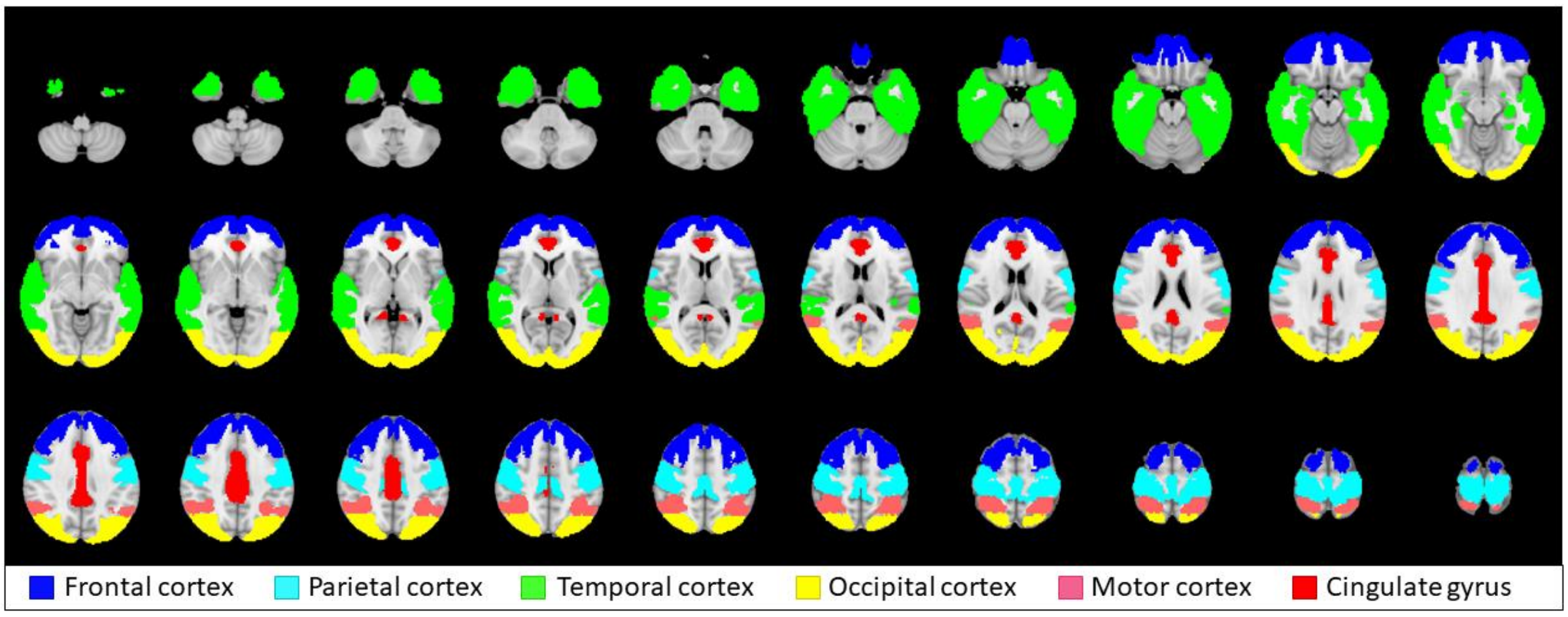

Figure S4: Grey matter masks used for region of interest analysis in MNI space.

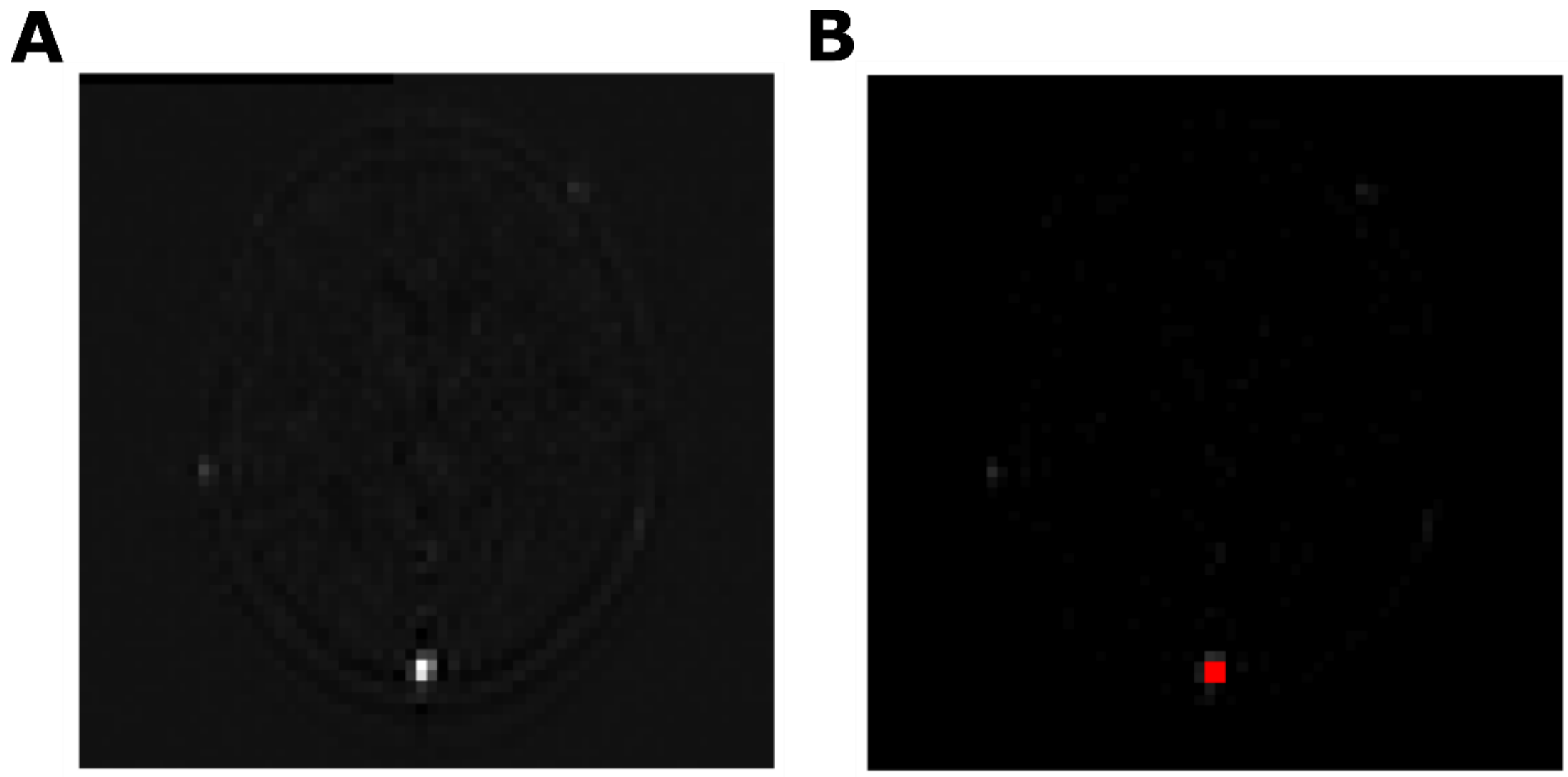

Figure S5: Exemplar images produced by the T_2-relaxation-under-spin-tagging (TRUST) MATLAB processing pipeline (n = 1). A: Averaged difference images from all echo times showing the highest signal in the sagittal sinus. B: Automatic identification of the four highest signal voxels within the sagittal sinus used for venous blood oxygen saturation estimates.

3) Results

Cardiorespiratory fitness: between-group differences

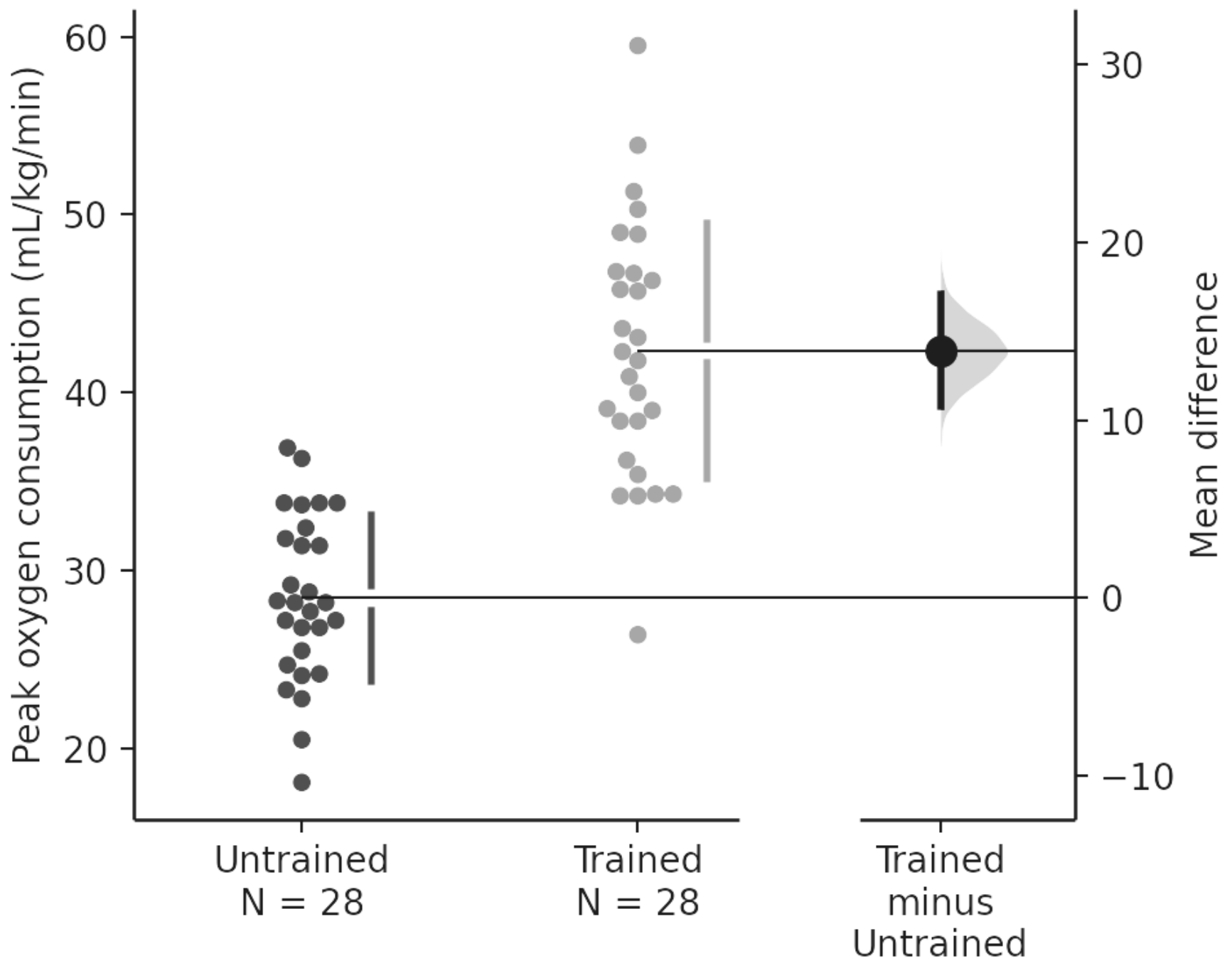

Figure S6: Individual participant data for each group including group mean and standard deviation, and the mean between-group difference with 95% confidence intervals in peak oxygen consumption ($\dot{V}O_{2peak}$) between untrained and trained older adults.

Cerebral haemodynamics: between-group differences

Table S1:
Means and 95% confidence intervals for cerebral haemodynamics in grey matter at rest in untrained and trained older adults.

	Untrained	Trained
CBF (mL/100 g/min)		
Global	68.5 [62.6, 74.4]	74.0 [67.5, 80.6]
ACA territory	92.0 [82.6, 101.4]	107.8 [97.7, 117.9]
MCA territory	84.9 [77.2, 92.6]	95.3 [86.5, 104.1]
PCA territory	68.5 [62.3, 74.8]	68.8 [62.4, 74.9]
ATT (seconds)		
Global	1.39 [1.33, 1.46]	1.28 [1.20, 1.36]
ACA territory	1.32 [1.27, 1.38]	1.25 [1.17, 1.32]
MCA territory	1.29 [1.22, 1.35]	1.19 [1.12, 1.26]
PCA territory	1.44 [1.39, 1.50]	1.38 [1.30, 1.45]

n = 28 per group. CBF: cerebral blood flow, ATT: arterial transit time, ACA: anterior cerebral arteries, MCA: middle cerebral arteries, PCA: posterior cerebral arteries.

Global between-group difference analyses were repeated whilst controlling for the potential effects of educational attainment. Using regressed data, two-sided permutation tests found mean group differences of 7.2 [-0.4, 15.5] mL/100 g/min ($P = 0.078$) and -0.12 [-0.22, -0.03] s ($P = 0.017$) for CBF and ATT, respectively. Similar to the primary analyses, an ANCOVA also found a significant effect of group on global ATT ($F(1, 51) = 4.92$, $P = 0.031$) when controlling for education ($F(3, 51) = 1.44$, $P = 0.242$).

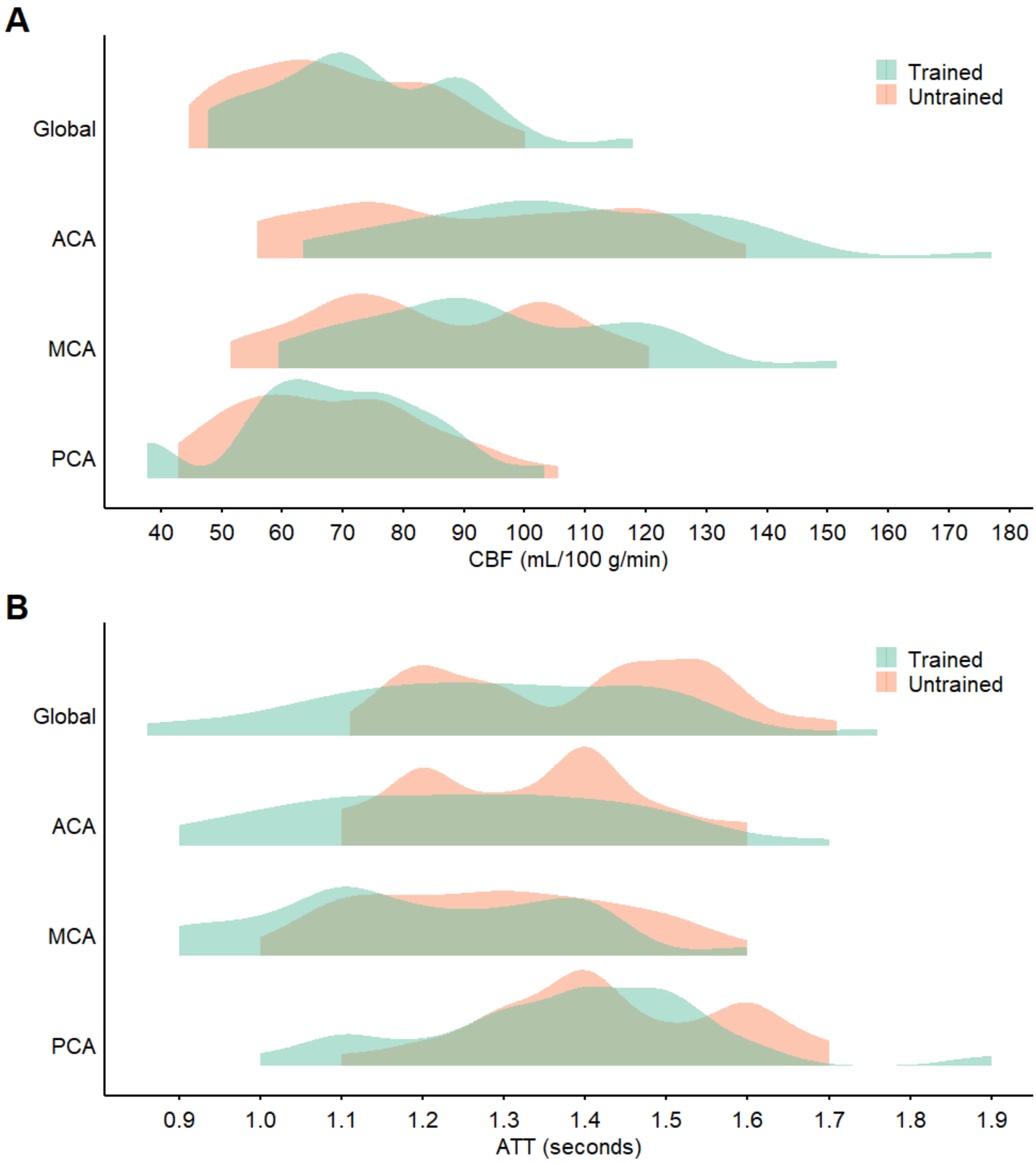

Figure S7: Distribution of global and regional territory data for A: grey matter cerebral blood flow (CBF) and B: grey matter arterial transit time (ATT) in untrained (*n* = 28) and trained (*n* = 28) participants. ACA: anterior cerebral arteries, MCA: middle cerebral arteries, PCA: posterior cerebral arteries.

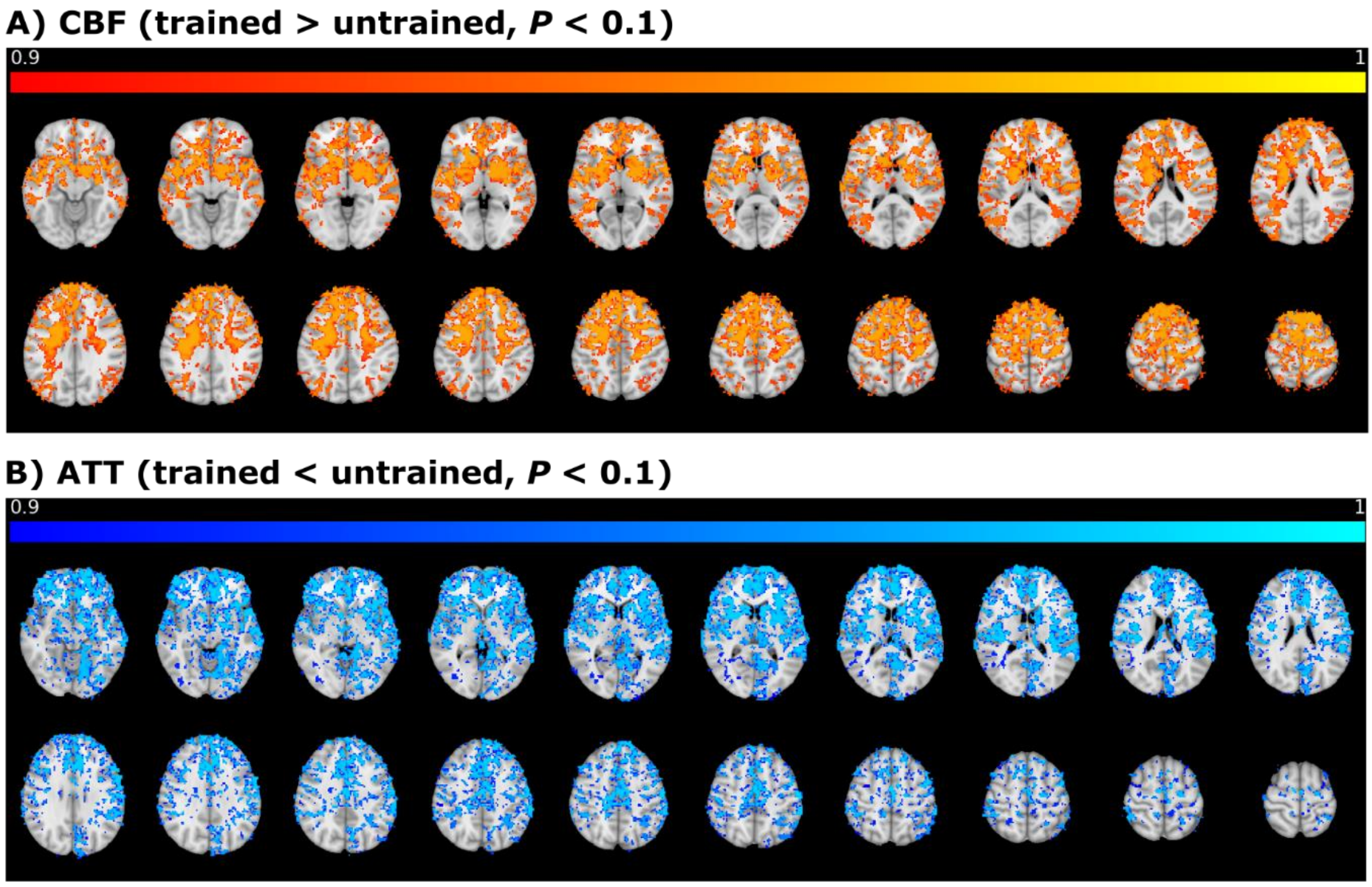

Figure S8: Liberal statistically thresholded maps of between-group differences in A: cerebral blood flow (CBF) and B: arterial transit time (ATT) (n = 28 per group). Only voxels with data from at least 80% of participants in each group were analysed. For CBF, red-yellow voxels indicate where grey matter CBF is greater in trained than untrained participants ($P < 0.1$) - all voxels where CBF is lower in trained than untrained participants, $P > 0.1$. For ATT, blue voxels indicate where grey matter ATT is shorter in trained than untrained participants ($P < 0.1$) - all voxels where ATT is longer in trained than untrained participants, $P > 0.1$. Analysed using *randomise* in FSL (threshold-free cluster enhancement applied, 5000 permutations).

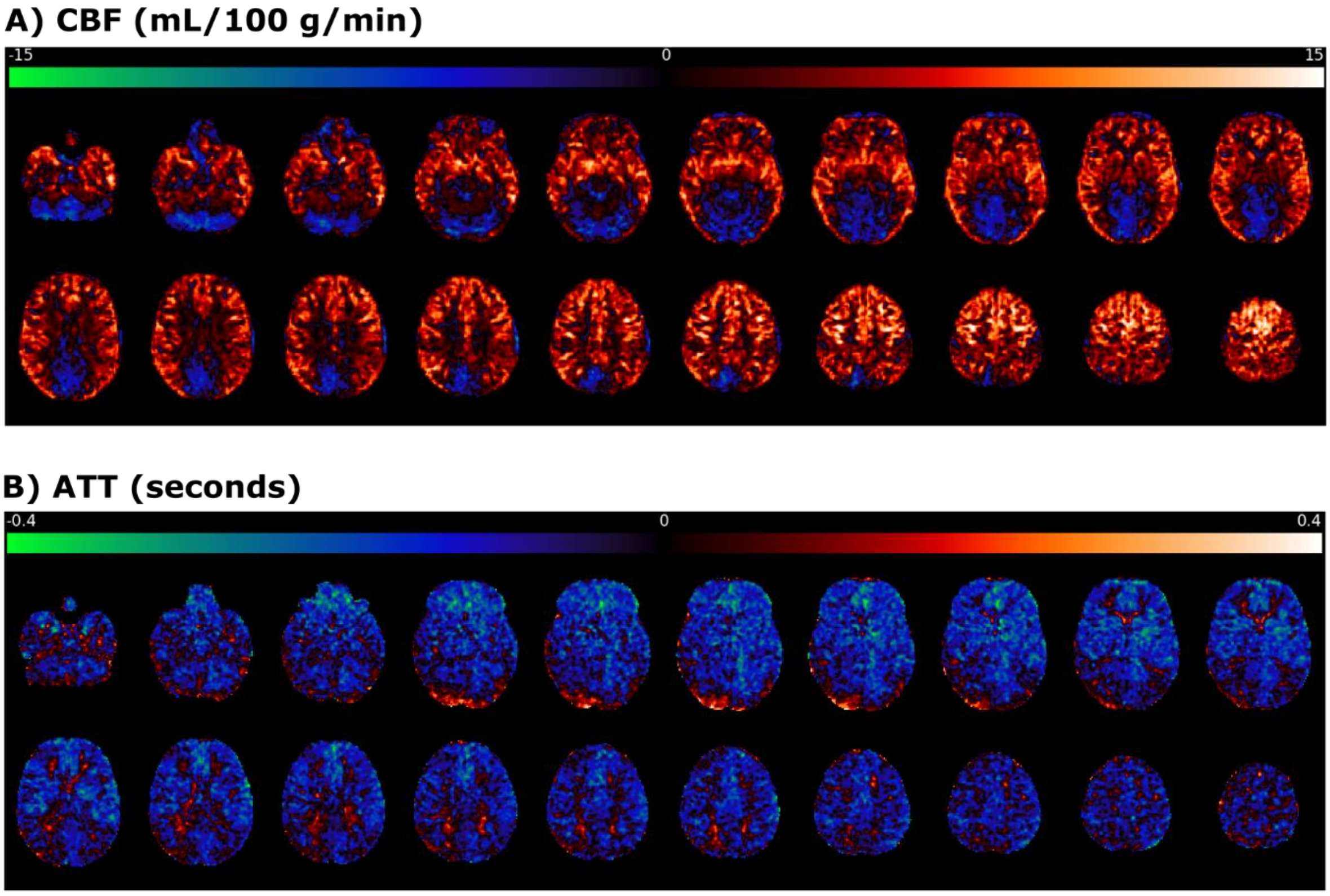

Figure S9: Descriptive brain maps of between-group differences in grey matter (before partial volume correction) A: cerebral blood flow (CBF) and B: arterial transit time (ATT). Calculated as: trained – untrained (*n* = 28 per group). Voxels with ≥80% participant data in each group are shown.

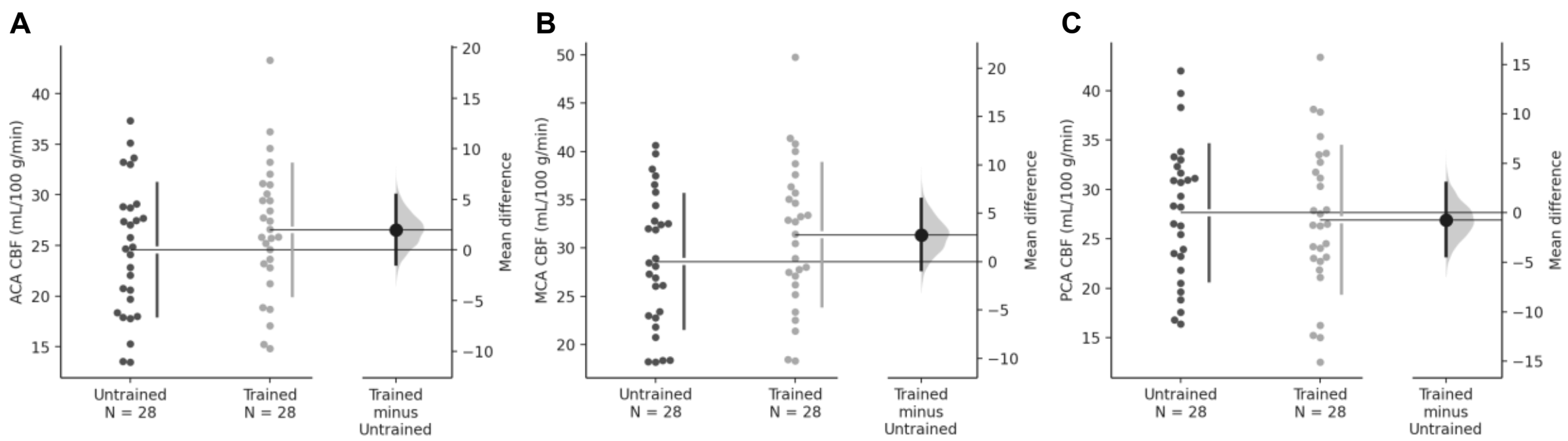

Figure S10: Individual participant data for each group including group mean and standard deviation, and the mean between-group difference with 95% confidence intervals in white matter cerebral blood flow (CBF) within regional vascular territories at rest between untrained and trained older adults. A: anterior cerebral arteries (ACA) territory, B: middle cerebral arteries (MCA) territory, C: posterior cerebral arteries (PCA) territory.

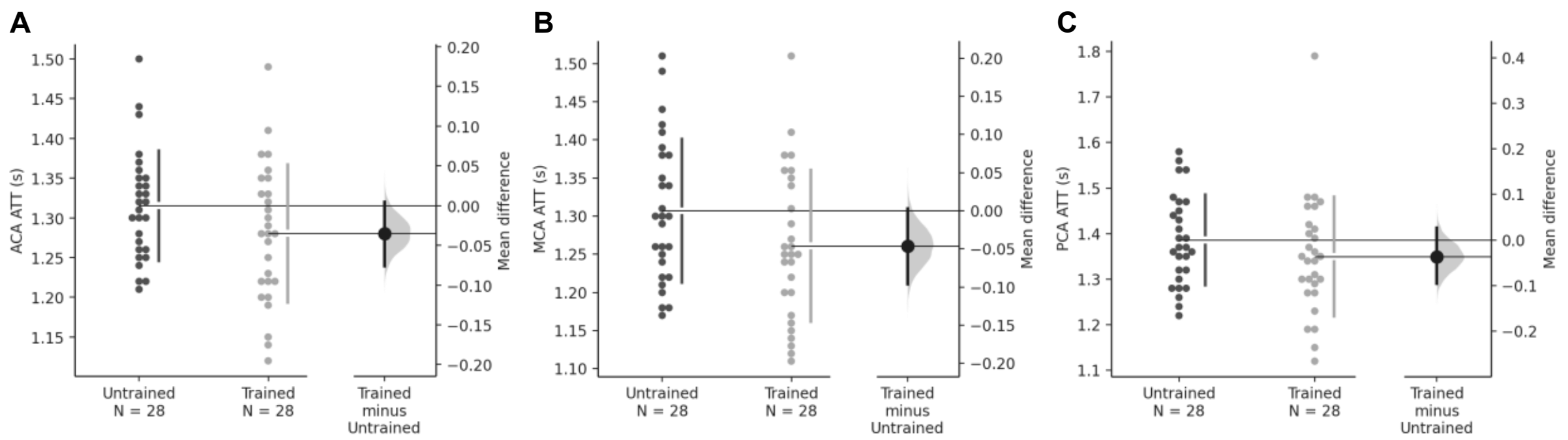

Figure S11: Individual participant data for each group including group mean and standard deviation, and the mean between-group difference with 95% confidence intervals in white matter arterial transit time (ATT) within regional vascular territories at rest between untrained and trained older adults. A: anterior cerebral arteries (ACA) territory, B: middle cerebral arteries (MCA) territory, C: posterior cerebral arteries (PCA) territory.

Secondary regional CBF analyses (Figure S8 and Table S2) identified the largest differences with medium effect sizes in frontal (12.1 [0.2, 24.1] mL/100 g/min, Hedges' *g* = 0.51 [-0.03, 1.04]), temporal (8.2 [-0.6, 17.4] mL/100 g/min, Hedges' *g* = 0.46 [-0.07, 0.97]), and motor (14.2 [0.3, 28.4] mL/100 g/min, Hedges' *g* = 0.51 [-0.01, 1.03]) regions, although these were not statistically significant ($P > 0.05$).

Secondary regional ATT analyses (Figure S9 and Table S2) identified the largest differences with medium effect sizes in frontal (-0.10 [-0.19, -0.01] s, Hedges' *g* = 0.56 [0.05, 1.09]) and cingulate (-0.12 [-0.21, -0.02] s, Hedges' *g* = 0.63 [0.09, 1.17]) regions, although these were not statistically significant after adjustment for multiple comparisons ($P > 0.05$).

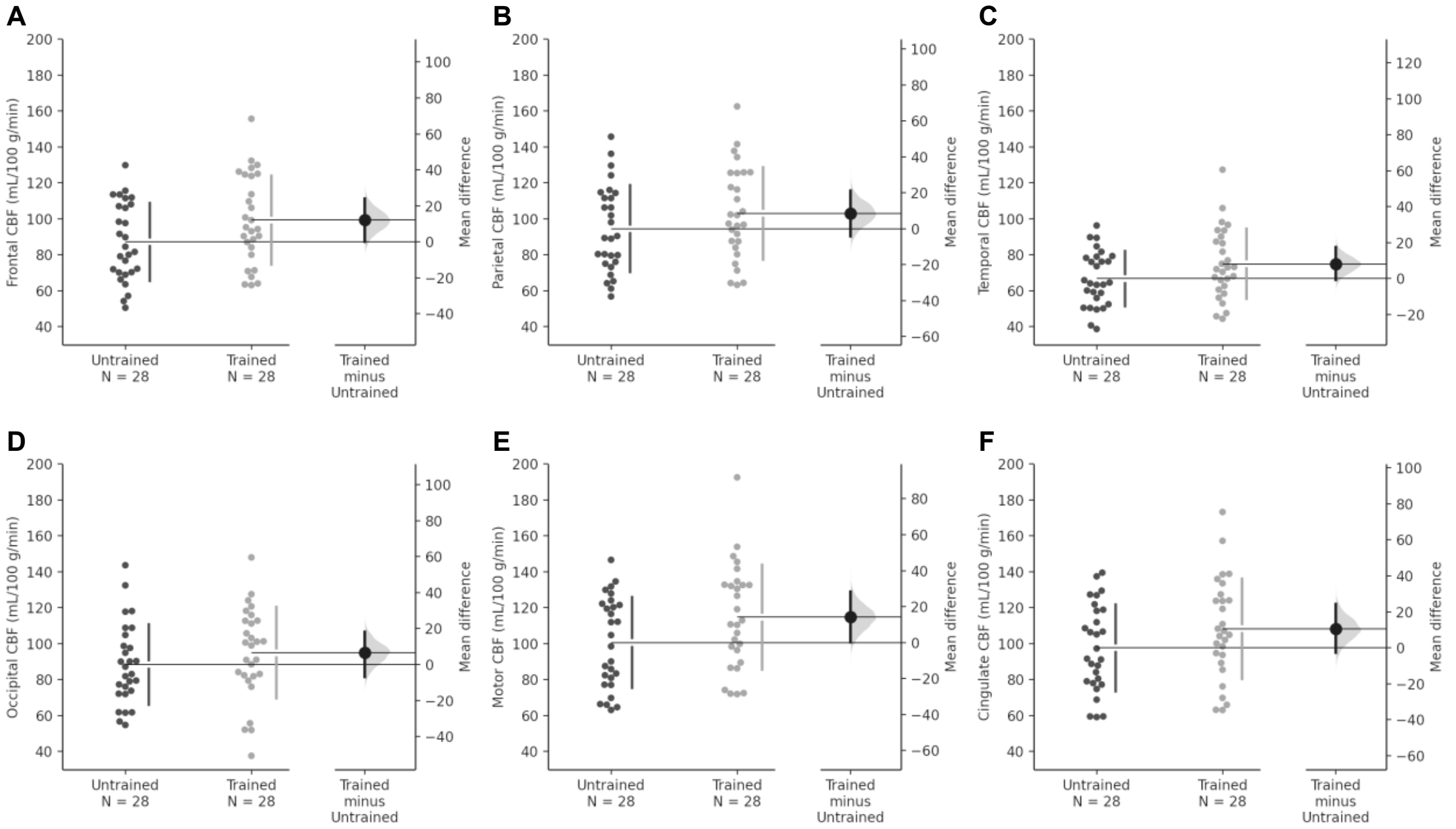

Figure S12: Individual participant data for each group including group mean and standard deviation, and the mean between-group difference with 95% confidence intervals in regional cerebral blood flow (CBF) in grey matter at rest between untrained and trained older adults. A: Frontal, B: Parietal, C: Temporal, D: Occipital, E: Motor, F: Cingulate.

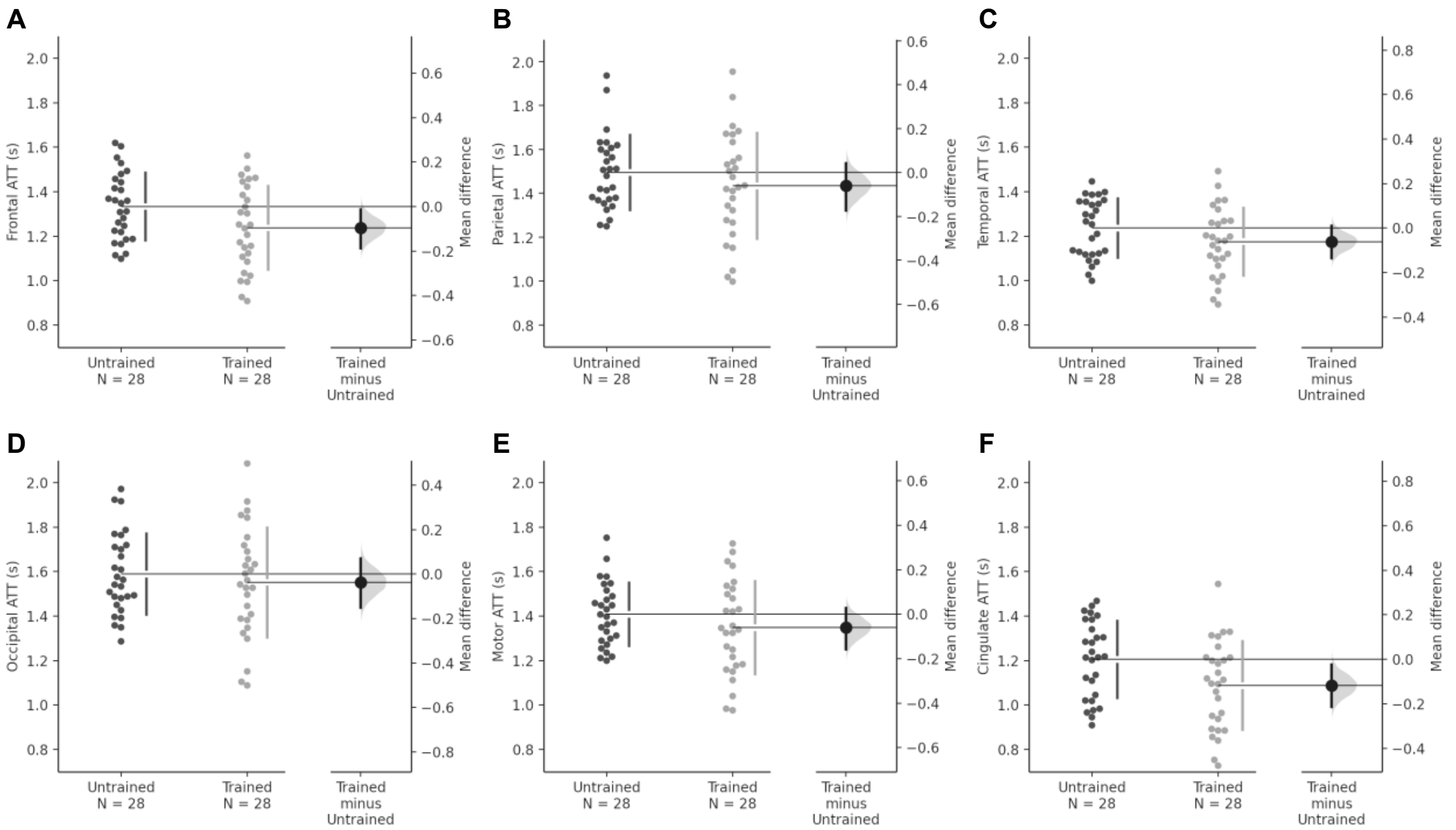

Figure S13: Individual participant data for each group including group mean and standard deviation, and the mean between-group difference with 95% confidence intervals in regional arterial transit time (ATT) in grey matter at rest between untrained and trained older adults. A: Frontal, B: Parietal, C: Temporal, D: Occipital, E: Motor, F: Cingulate.

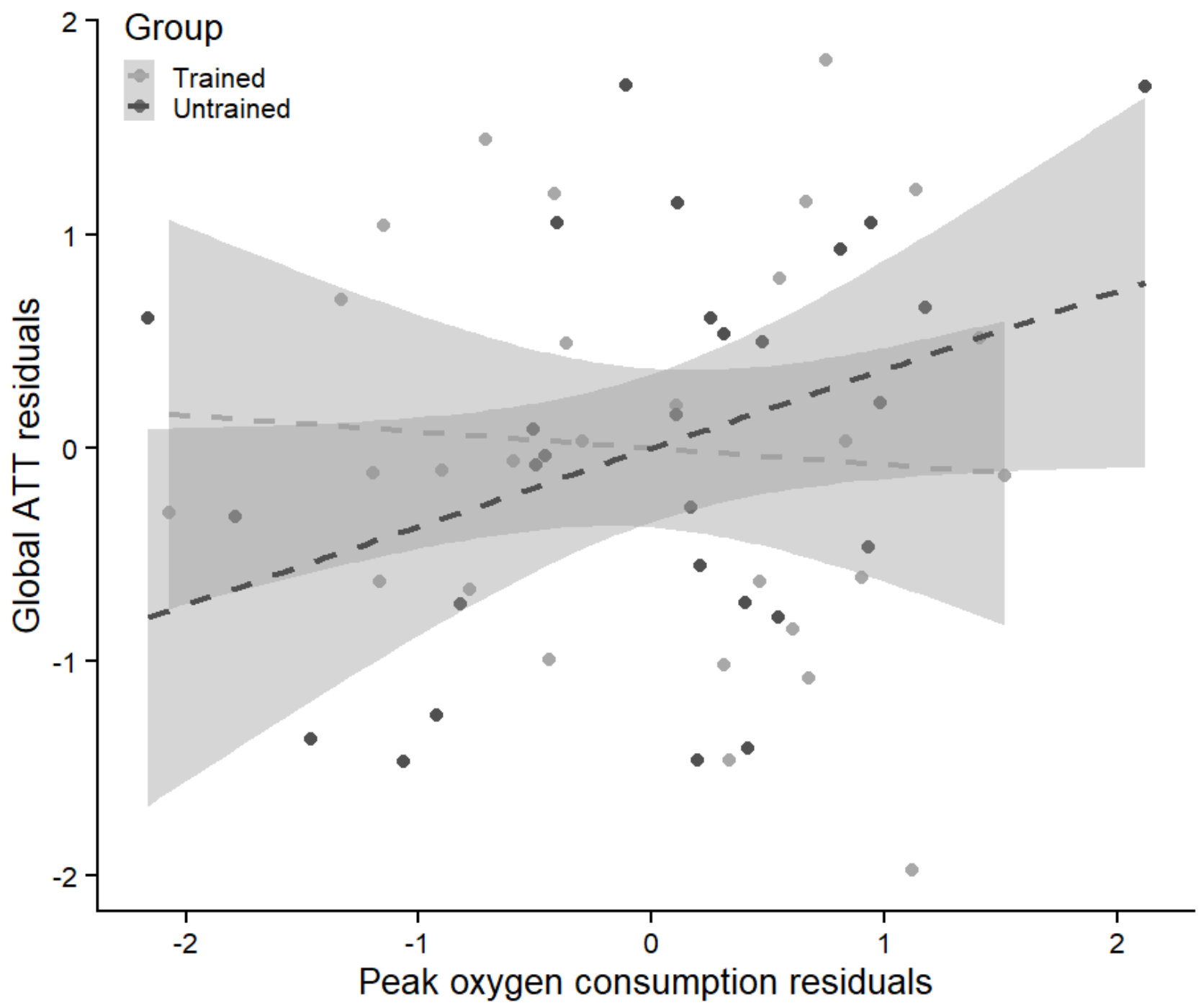

Figure S14: Partial regression associations between peak oxygen consumption and grey matter global arterial transit time (ATT) in untrained (dark grey, *n* = 28) and trained (light grey, *n* =28). Untrained: β = 0.79 [-0.07, 1.65], *P* = 0.071. Trained: β = -0.19 [-1.26, 0.88], *P* = 0.715. Model: ATT ~ age + sex + BMI + $\dot{V}O_{2peak}$. BMI: body mass index, $\dot{V}O_{2peak}$: peak oxygen consumption.

Table S2:
Between-group differences in regional cerebral haemodynamics in grey matter at rest.

	Mean difference	Hedges' *g*	*P*
CBF (mL/100 g/min)			
Frontal	12.1 [0.6, 24.1]	0.51 [-0.03, 1.04]	0.058
Parietal	8.6 [-4.2, 21.3]	0.34 [-0.20, 0.88]	0.205
Temporal	8.2 [-0.6, 17.4]	0.46 [-0.07, 0.97]	0.084
Occipital	6.5 [-7.0, 18.1]	0.27 [-0.30, 0.83]	0.314
Motor	14.2 [0.31, 28.4]	0.51 [-0.01, 1.03]	0.055
Cingulate	10.6 [-2.6, 24.3]	0.40 [-0.13, 0.95]	0.133
ATT (seconds)			
Frontal	-0.10 [-0.19, -0.01]	-0.56 [-1.09, -0.05]	0.041
Parietal	-0.06 [-0.17, 0.04]	-0.29 [-0.82, 0.23]	0.291
Temporal	-0.06 [-0.13, 0.01]	-0.42 [-0.96, 0.10]	0.114
Occipital	-0.04 [-0.15, 0.07]	-0.17 [-0.70, 0.35]	0.532
Motor	-0.06 [-0.16, 0.03]	-0.33 [-0.86, 0.19]	0.219
Cingulate	-0.12 [-0.21, -0.02]	-0.63 [-1.17, -0.09]	0.021

Mean difference (trained – untrained), *Hedges' g with 95% confidence intervals, and unadjusted regional P values. ATT: arterial transit time, CBF: cerebral blood flow.*

Cognitive functions: data quality

Working memory (2-back): No correct trials were excluded for slow or fast reaction times. Correct response reaction times were 633 ± 171 ms and 566 ± 175 ms for untrained and trained groups, respectively. Percentage of correct hit trials (*n* = 12) were 81 ± 18 % and 79 ± 23 % for untrained and trained groups, respectively. Percentage of correct rejections trials (*n* = 48) were 95 ± 4 % and 95 ± 4 % for untrained and trained groups, respectively.

Attention Network Task: Of the total 288 trials, 97 ± 6 % and 98 ± 2 % were responded to correctly by untrained and trained groups, respectively. Of these correct trials, 5 ± 1 % and 4 ± 1 % were excluded from analysis for slow or fast reaction times for untrained and trained groups, respectively. Correct response reaction times were 682 ± 90 ms and 652 ± 69 ms for untrained and trained groups, respectively.

Cognitive functions: between-group differences

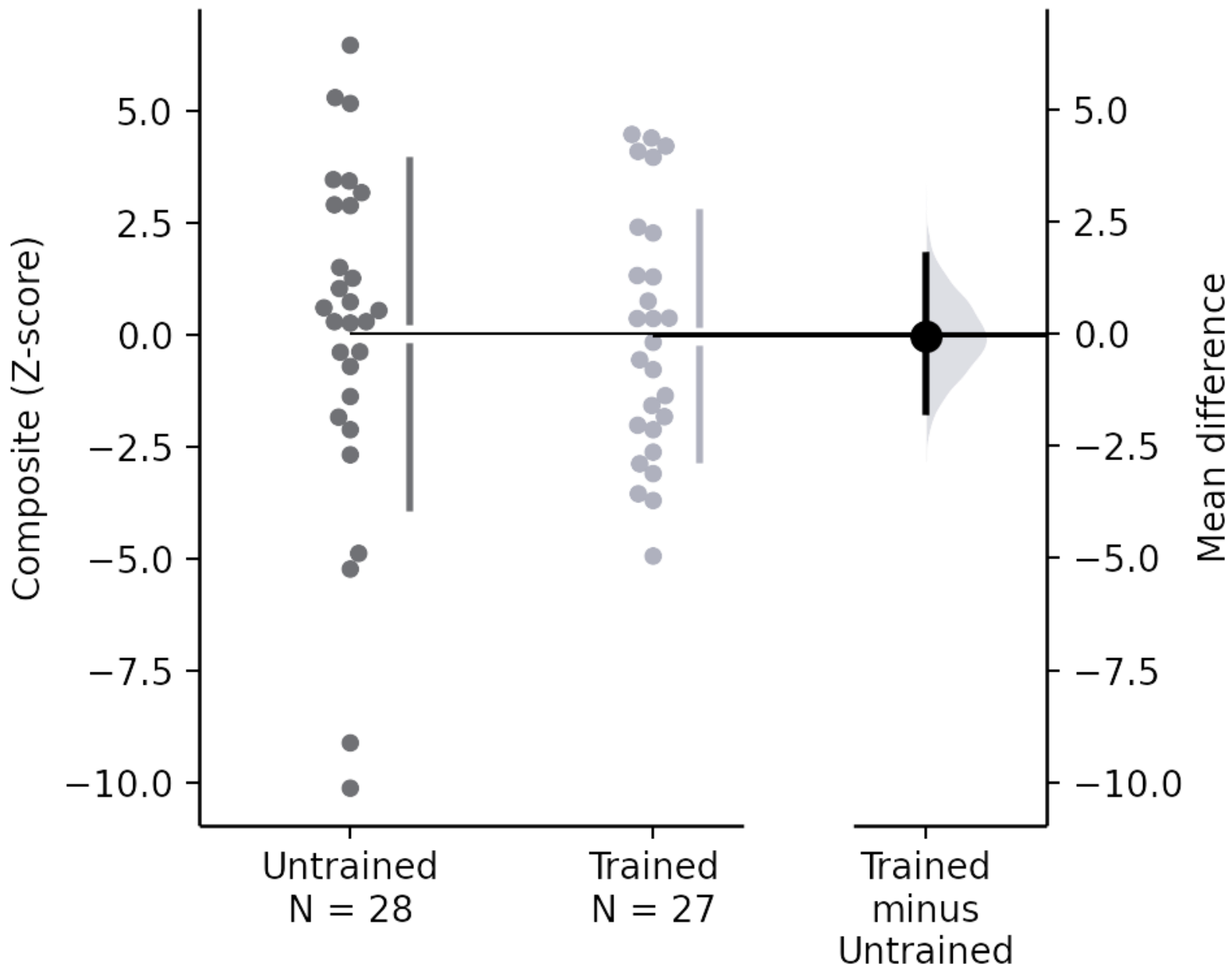

Figure S15: Individual participant data for each group including group mean and standard deviation, and the mean between-group difference with 95% confidence intervals in composite cognitive function Z-scores between untrained and trained older adults.

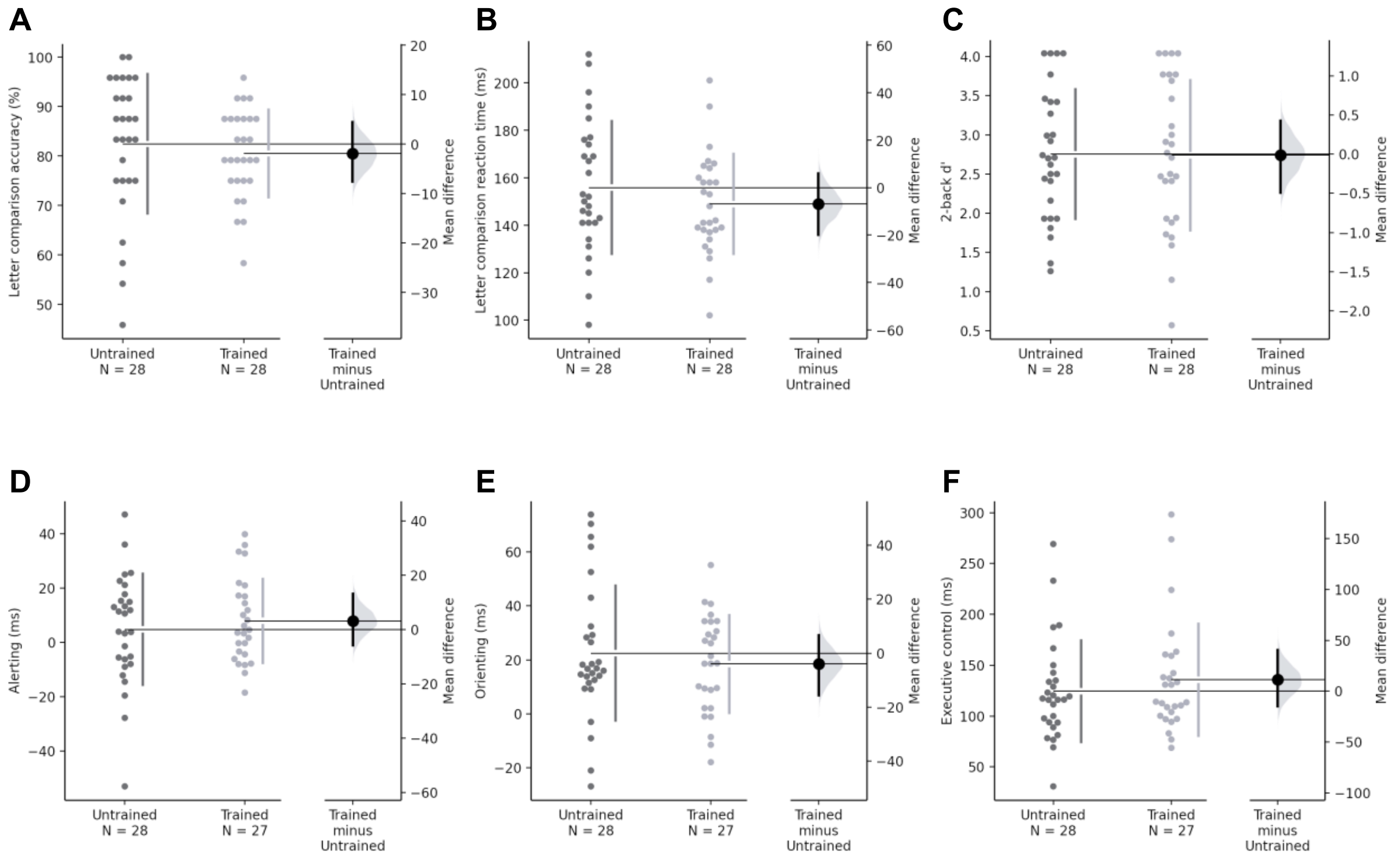

Figure S16: Individual participant data for each group including group mean and standard deviation, and the mean between-group difference with 95% confidence intervals in processing speed (A, B), working memory (C), and attention (D – F) between untrained and trained older adults.

Cerebral haemodynamics and cerebral oxygen extraction fraction: associations

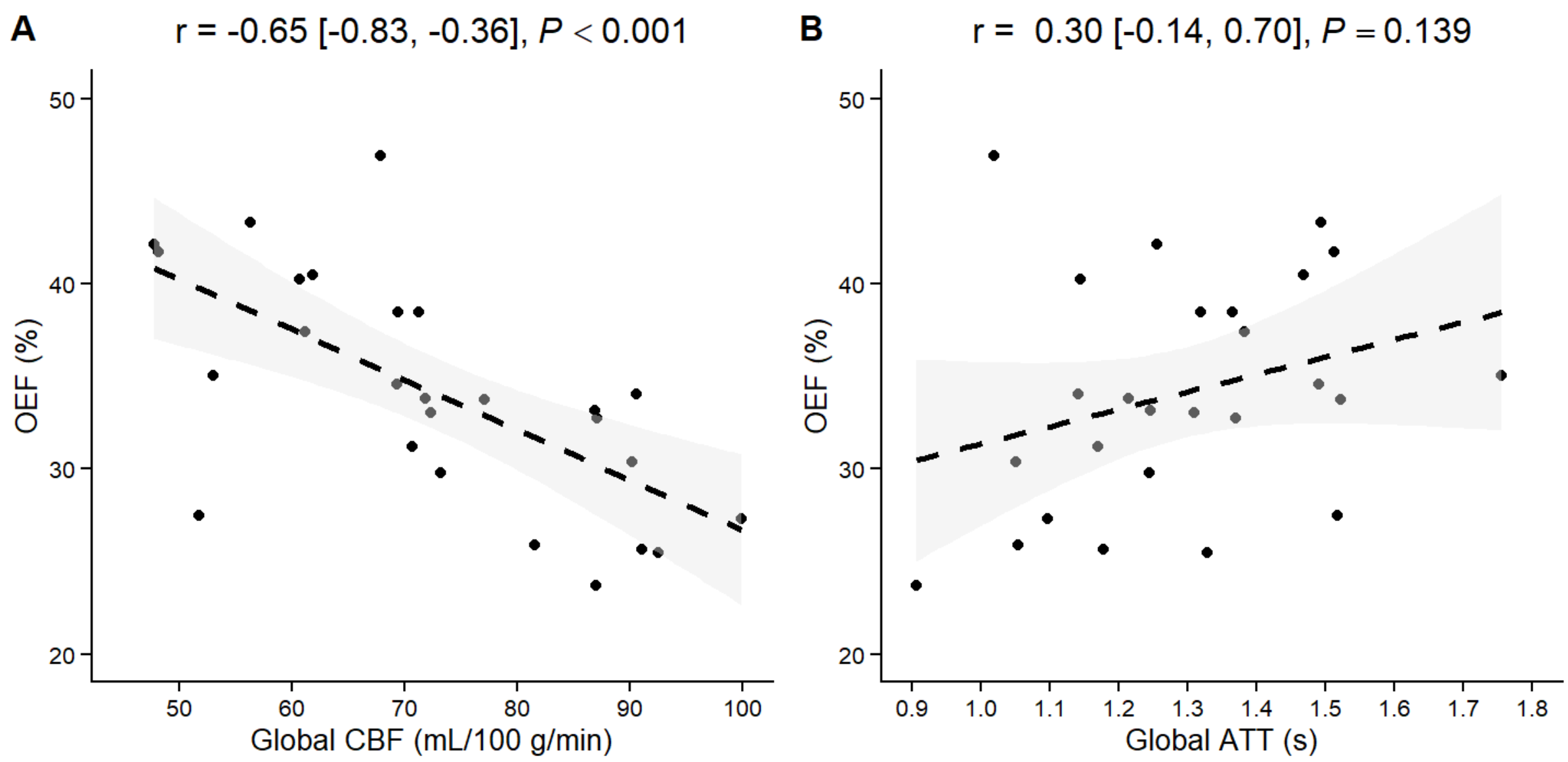

Figure S17: Bivariate Pearson correlations between cerebral oxygen extraction fraction (OEF) and A: global grey matter cerebral blood flow (CBF), or B: global grey matter arterial transit time (ATT) in trained participants (n = 26).

ANT cue types

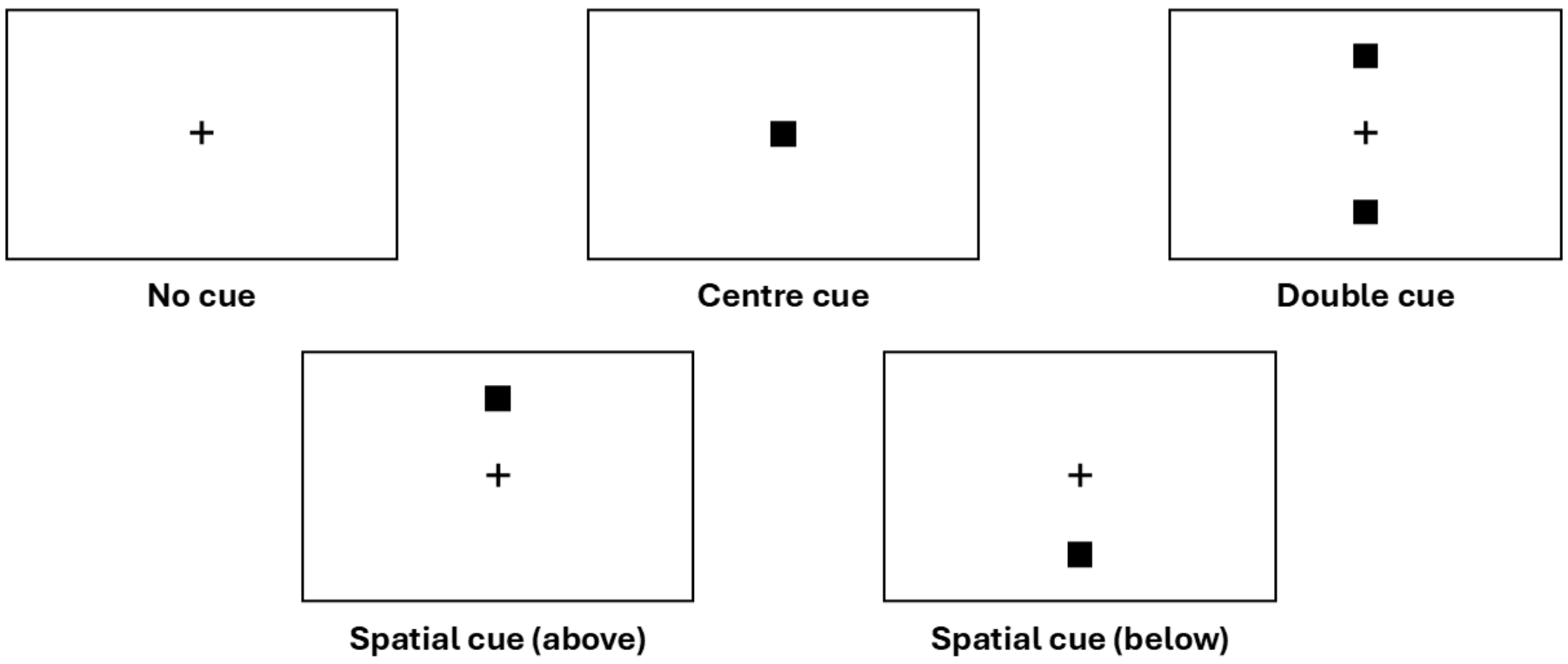

ANT trial types

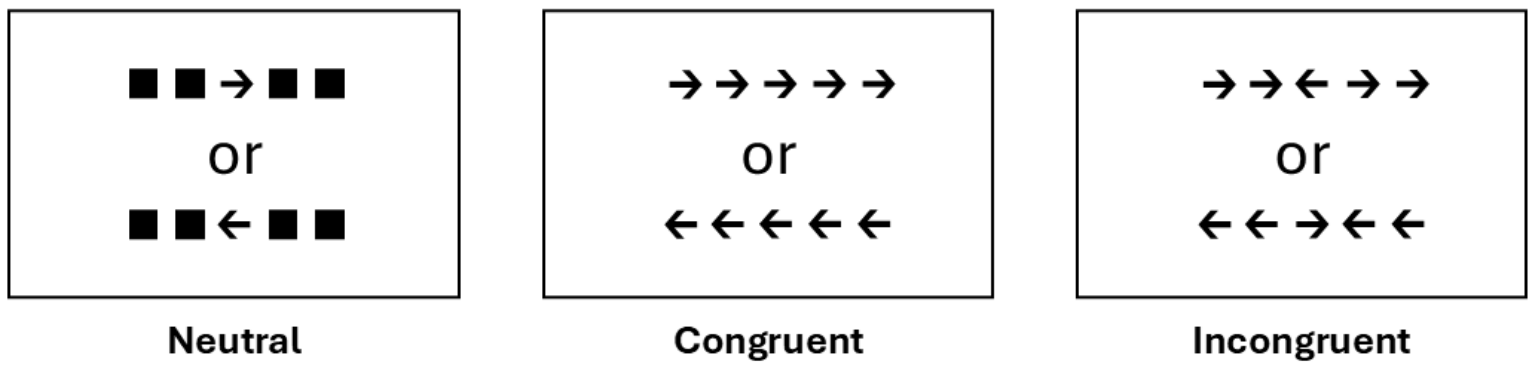

Only one stimulus is shown per trial. The stimulus is located either above or below the central fixation cross. The location of the stimulus is cued only by the spatial cues (above or below). For all other cues, the stimulus could appear above or below the central fixation cross.

ANT condition difference scores

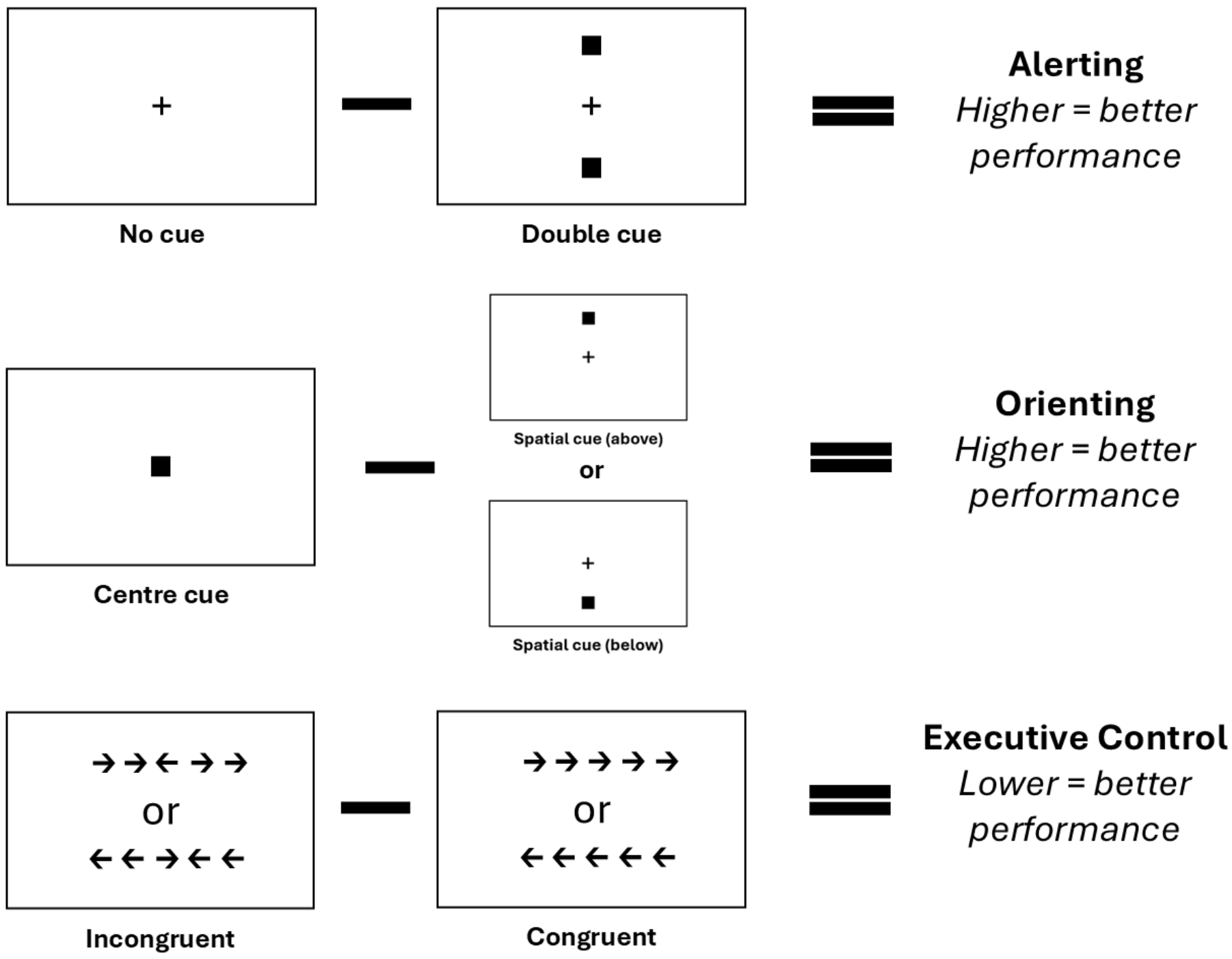

Figure S18: Visualisation of the attentional network task (ANT) cue types and trial conditions, and depiction of how the three outcome measures are calculated.

References

Burley, C.V., Francis, S.T., Whittaker, A.C., Mullinger, K.J., Lucas, S.J.E., 2021. Measuring resting cerebral haemodynamics using MRI arterial spin labelling and transcranial Doppler ultrasound: Comparison in younger and older adults. Brain and Behavior 11, e02126. https://doi.org/10.1002/brb3.2126

Carson, N., Leach, L., Murphy, K.J., 2018. A re-examination of Montreal Cognitive Assessment (MoCA) cutoff scores. Int J Geriatr Psychiatry 33, 379–388. https://doi.org/10.1002/gps.4756

Feron, J., Rahman, F., Fosstveit, S.H., Joyce, K.E., Gilani, A., Lohne-Seiler, H., Berntsen, S., Mullinger, K.J., Segaert, K., Lucas, S.J.E., 2024a. Cerebral blood flow and arterial transit time responses to exercise training in older adults. NeuroImage 303, 120919. https://doi.org/10.1016/j.neuroimage.2024.120919

Feron, J., Segaert, K., Rahman, F., Fosstveit, S.H., Joyce, K.E., Gilani, A., Lohne-Seiler, H., Berntsen, S., Mullinger, K.J., Lucas, S.J.E., 2024b. Determinants of cerebral blood flow and arterial transit time in healthy older adults. Aging (Albany NY). https://doi.org/10.18632/aging.206112

Liu, C.-F., Hsu, J., Xu, X., Kim, G., Sheppard, S.M., Meier, E.L., Miller, M.I., Hillis, A.E., Faria, A.V., 2023. Digital 3D Brain MRI Arterial Territories Atlas. Sci Data 10, 74. https://doi.org/10.1038/s41597-022-01923-0
